# Health-related quality of life in recessive dystrophic epidermolysis bullosa: findings of the Prospective Epidermolysis Bullosa Longitudinal Evaluation Study (PEBLES)

**DOI:** 10.1186/s13023-026-04330-5

**Published:** 2026-05-06

**Authors:** Eunice Jeffs, Elizabeth I. Pillay, Lesedi Ledwaba-Chapman, Alessandra Bisquera, Susan J. Robertson, John A. McGrath, Yanzhong Wang, Anna E. Martinez, Jemima E. Mellerio

**Affiliations:** 1 St John’s Institute of Dermatology, Guy’s and St Thomas’ NHS Foundation Trust, London, UK; 2 Department of Population Health Sciences, King’s College London, London, UK; 3 Departments of Dermatology, Department of Paediatrics, The Royal Children’s Hospital, The Royal Melbourne Hospital and Murdoch Children’s Research Institute, University of Melbourne, Melbourne, Australia; 4 St John’s Institute of Dermatology, King’s College London, London, UK; 5 Department of Dermatology, Great Ormond Street Hospital for Children NHS Foundation Trust, London, UK

**Keywords:** Epidermolysis bullosa, Health-related quality of life, Disease severity, Natural history

## Abstract

**Background:**

Chronic disease, including different forms of epidermolysis bullosa (EB), may significantly impair health-related quality of life (HRQoL). To date, HRQoL in specific subtypes of recessive dystrophic EB (RDEB) has not been studied in depth.

**Objectives:**

To measure HRQoL in a large cohort of individuals with different RDEB subtypes, to explore differences in physical functioning and emotional/psychosocial health scores, and to identify potential correlation with disease severity.

**Methods:**

The Prospective EB Longitudinal Evaluation Study (PEBLES) is a register study of children and adults with RDEB. Reviews are repeated every 6 months (under 10 years) or annually (10 years and above) with HRQoL assessed using the Quality of Life in Epidermolysis Bullosa (QOLEB), an EB-specific questionnaire, for adult participants and the Pediatric Quality of Life Inventory (PedsQL) generic core scales, version 4.0, for child participants and their parents. Disease severity was measured with the Birmingham EB Severity score (BEBS) and the Instrument for Scoring Clinical Outcomes for EB (iscorEB).

**Results:**

HRQoL was reported in 335 reviews over a maximum of seven years by 61 participants: severe RDEB (RDEB-S) *n* = 26, intermediate (RDEB-I) *n* = 21, inversa (RDEB-Inv) *n* = 9, pruriginosa (RDEB-Pru) *n* = 4 and pretibial RDEB *n* = 1. QOLEB demonstrated a severe impact on HRQoL for all RDEB adults (*n* = 47), particularly for RDEB-Pru and RDEB-S participants. Total and functioning QOLEB scores correlated with disease severity scores (iscorEB, BEBS) for all RDEB, with a statistically higher impact in RDEB-S compared to RDEB-I and RDEB-Inv. In children (*n* = 14), those with greater disease severity measured by iscorEB also reported worse HRQoL (PedsQL). In adults and children, physical functioning/health QoL was more severely impacted than emotions/psychosocial health, and HRQoL generally improved with age.

**Conclusion:**

Our results highlight a significant impact on HRQoL in adults and children with all types of RDEB which generally correlates with disease severity. Relatively less impact on emotional functioning/psychosocial health rather than physical functioning/health scores suggests psychological adaptation from living with RDEB, a lifelong condition which typically presents at or shortly after birth. Further, a relative improvement in HRQoL with age, despite disease progression and increasing severity over time, supports ongoing adaptation throughout life.

**Supplementary Information:**

The online version contains supplementary material available at 10.1186/s13023-026-04330-5.

## Background

Epidermolysis bullosa (EB) is a heterogeneous group of rare inherited disorders whose principal feature is mucocutaneous fragility [1]. Extracutaneous features and disease severity vary between and within each type, as does the impact on health-related quality of life (HRQoL) of affected individuals [2, 3]. Whilst milder EB forms have limited skin blistering and a normal lifespan, other variants, such as recessive dystrophic EB (RDEB), can have widespread skin and systemic involvement, and foreshortened life expectancy [4, 5].

All forms of dystrophic EB (DEB) result from pathogenic variants in the *COL7A1* gene, encoding type VII collagen, which forms anchoring fibrils at the dermal-epidermal junction [6]. RDEB is inherited as an autosomal recessive trait, with distinct subtypes differentiated by clinical and molecular features [6, 7]. In severe RDEB (RDEB-S), extensive mucocutaneous blistering result in chronic wounds, scarring, corneal erosions, oesophageal strictures, hand contractions and mitten deformities, with systemic sequelae including anaemia, nutritional compromise and osteoporosis. Severe pain and pruritus are common, wound infections a constant hazard, and from adolescence onwards there is a high incidence of aggressive cutaneous squamous cell carcinomas (SCC) which frequently cause death in early adulthood [4, 5, 8, 9]. Intermediate RDEB (RDEB-I) also has generalized skin fragility, but the extent and severity are less marked, and SCCs are less common and occur later in life [4, 8, 9]. In RDEB inversa (RDEB-Inv), skin and mucosal involvement are not usually particularly severe in childhood but subsequently a predilection develops for flexural skin and mucosal involvement, particularly of the mouth, oesophagus and vulva [10]. Similarly, RDEB pruriginosa (RDEB-Pru) tends to initially present as a milder form although, around adolescence, extreme skin itching develops with prurigo-like lesions and linear scarring particularly on the limbs [11]. Both RDEB-Pru and RDEB-Inv may be initially diagnosed as RDEB-I. In localised RDEB, skin involvement tends to be in limited areas and relatively mild, typically pretibial (RDEB-PT) [1].

The complex health problems in RDEB significantly impact daily life and function: pain and pruritus disturb sleep; scarring and pain affect mobility, dexterity and independence; wound care and dressing changes require many hours each week for both affected individuals and their carers [12–17]. The psychological and emotional impact of RDEB may impair social relationships and limit life opportunities, with a negative effect on an individual’s participation in education and employment [16, 18, 19]. Family members who act as unpaid carers may also have limited employment opportunities [16, 20].

RDEB is a very visible rare disease, poorly understood at a societal level and which can leave individuals and families feeling isolated [13, 19, 21]. In severe forms, dependence on others, poor health, anxiety of having a life-limiting illness, and a constant need for vigilance for SCCs all add to the burden of living with RDEB [13, 14, 22, 23]. Even less severe forms of EB can be burdensome, with evidence suggesting greater DEB disease severity correlates with a more negative impact on HRQoL [18, 23–26]. However, HRQoL in all the different subtypes of RDEB has not previously been explored in detail despite the wide variation in clinical presentation and severity.

The World Health Organization 1948 definition of health included the presence of physical, psychological and social wellbeing as well as the absence of disease and infirmity [27]. Increasingly, HRQoL, which incorporates objective assessment of physical, mental and social status and subjective perceptions and expectations, has become a key concept in healthcare practice and research in the assessment of an individual’s ability to lead a fulfilling life [28]. In research, HRQoL is an accepted patient-related outcome which reflects the impact of health or disease on an individual’s functioning and a key tool to compare patient groups and monitor wellbeing over time.

Various tools have been used to measure QoL and HRQoL in EB. Generic tools such as EuroQol 5 dimensions (EQ-5D) [29] are short and easy to use, enabling comparisons across a broad range of health conditions. EQ-5D has been used in a few EB studies, demonstrating a negative impact of disease on HRQoL [20, 23, 30]. Dermatology-specific measures such as the Dermatology Life Quality Index (DLQI) and the Children’s Dermatology Life Quality Index (CDLQI) have been widely used in skin disease research [31, 32] but may exhibit a ceiling effect for more severely impacted individuals such as EB, where the severity and breadth of symptoms may be very profound, raising concerns about content validity. The Quality of Life in Epidermolysis Bullosa (QOLEB) questionnaire was devised to be relevant for people living with EB [33]. It has been translated into and validated for a number of languages [33–41] and is the most used tool for evaluating EB-related HRQoL. However, although differences within and between the major types of EB have been relatively well studied [3, 34, 42–47], exploration of differences within RDEB has been restricted to small numbers and limited specific RDEB subtypes [3, 34].

The Prospective Epidermolysis Bullosa Longitudinal Evaluation Study (PEBLES) is an ongoing prospective register study capturing the natural history of RDEB. Here, we report HRQoL of individuals with different RDEB subtypes including their perceptions of their health and how these impact on their physical functioning and emotional or psychosocial health state.

## Methods

### Study population

PEBLES participants were recruited from individuals attending the London, UK, EB centres (Great Ormond Street Hospital for Children (children) and Guy’s and St Thomas’ Hospital (adults)) between 19th November 2014 and 17th November 2021. All had a confirmed diagnosis of RDEB by skin biopsy and/or genetic testing, with RDEB subtype classified according to clinical features. Participants provided comprehensive data at baseline and subsequent reviews, including EB- and non-EB-related health issues, data regarding all body systems and management of RDEB, disease severity scores, utilisation of hospital and community care, and self-reported itch, pain and HRQoL. The researcher updated information since the preceding visit, annually (10-year-olds and above) or 6-monthly (under-10s), through face-to-face interviews and self-reported questionnaires, with virtual participant reviews (telephone or video call) conducted during the Covid-19 pandemic. Routine patient care was maintained by clinical teams at each site. PEBLES was ethically approved by the UK Research Ethics Committee London-Bromley and Health Research Authority (IRAS 142032).

### Measures

HRQoL was assessed in adults (19 years and above) using the 17-item QOLEB questionnaire validated for adults with EB [33] and in children (2–18 years) using the 23-item Pediatric Quality of Life Inventory (PedsQL), version 4.0 generic core scales [48], because there was no EB-specific QOL tool validated for use with children. QOLEB has a maximum score of 51, comprising 36 points allocated to functioning (sum of questions 1–7,9–10,12–13,15) and 15 points for emotions (sum of questions 8,11,14,16,17), with higher scores indicating worse HRQoL. Reported QOLEB cut offs for severity are: very mild 0–4 points, mild 5–9, moderate 10–19, severe 20–34 and very severe 35–51 [41]. Children aged 4–18 years completed PedsQL, and the parent or guardian of children aged 2–18 years completed companion questionnaires regarding their perceptions of their child’s health. PedsQL comprises 4 dimensions of physical, emotional, social and school functioning, with scores transformed into a total score ranging from 0 to 100; higher scores indicate better HRQoL. Two summary scores are calculated, each ranging from 0 to 100: physical health (8 items, same as physical functioning) and psychosocial health (15 items, including emotional, social and school functioning subscales). QOLEB and PedsQL scores with ≥ 1 missing response were excluded from the analysis.

Disease severity was measured using two of the validated EB-specific questionnaires available at commencement of PEBLES in 2014: Instrument for Scoring Clinical Outcomes for EB (iscorEB) [45, 47] and the Birmingham EB Severity score (BEBS) [49]. iscorEB contains five domains scored by clinicians (ISC, maximum score of 138) and 15 self-reported items completed by the patient or their carer (ISP, maximum score of 120); the total iscorEB score is the sum of valid ISC and ISP scores. The 11-item BEBS is scored by clinicians (maximum of 100). Higher scores for iscorEB and BEBS indicate greater RDEB activity/severity. BEBS and iscorEB scores with ≥ 1 missing response were excluded from the analysis.

All validated questionnaires were used with permission.

### Statistical analysis

Data were pseudonymised and recorded in a Research Electronic Data Capture (REDCap) database, retaining date of birth to link participants’ age to each review. To provide a snapshot of HRQoL for all RDEB and by subtype, findings are presented for (1) the index review, the first available review with complete HRQoL data, and (2) an average of per-participant HRQoL from all available reviews. Categorical variables are reported as counts and percentages, with continuous variables summarised using medians and inter-quartile range (IQR, that is (1st quartile, 3rd quartile)). Where appropriate, and not reported in the main text, results for all reviews are provided as supplementary material. Pairwise comparisons between RDEB subtypes for HRQoL parameters were computed using the Mann-Whitney U test, adjusted using the Benjamini-Hochberg procedure with a false discovery rate of 5%. Correlations between age, disease severity and HRQoL parameters were computed using Spearman’s rank correlation and interpreted as small, *r*=.10-0.29, medium, *r*=.30-0.49, or large, *r*=.50 − 1.0, according to Cohen [50]; these are reported only when 95% confidence intervals do not contain 0, indicating the correlation is significant (*p*<.05). All analyses were performed using R (version 4.1.3).

Missing-ness of data is reported where relevant in the tables and figures. One child-participant with RDEB-I was excluded from the analysis as parent questionnaires were incomplete and the child (aged 3.6 years) was too young to complete PedsQL. Data for the sole participant with RDEB-PT (5 reviews) were included only in overall analysis.

## Results

### Overview of data and demographics

HRQoL scores were available for a total of 335 reviews from 61 participants, with a median 6 (4,7) reviews from each participant over a median 6 (3,7) years; these included 26 individuals with RDEB-S (166 reviews), 21 with RDEB-I (99 reviews), 9 with RDEB-Inv (51 reviews), 4 with RDEB-Pru (14 reviews), and 1 with RDEB-PT (5 reviews). Table 1 shows participant demographics at index review. Table 2 shows symptom severity scores at index review, demonstrating substantially higher (worse) scores for individuals with RDEB-S and RDEB-Pru compared with intermediate and inversa subtypes. Average annual time spent doing dressings at index review was 332 (90,546) hours for all RDEB, and higher for RDEB-S at 520 (312–910) hours than for other subtypes. Supplementary material 1 provides severity scores for all reviews (*n* = 335).

**Table 1:** Demographics at index review

					RDEB Subtype			
Characteristic	Category	All RDEB	S		I	Inv	PT	Pru
n		**61**	**26**		**21**	**9**	**1**	**4**
Age, (years)		36 [23,48]	25 [9,33]		47 [38,64]	40 [30,48]	72 [72,72]	47 [38,57]
Age group, (years)	0 < 10	9 (15)	8 (31)		1 (5)	0 (0)	0 (0)	0 (0)
	10 < 18	5 (8)	3 (12)		2 (10)	0 (0)	0 (0)	0 (0)
	18 < 40	22 (36)	12 (46)		4 (19)	4 (44)	0 (0)	2 (50)
	≥ 40	25 (41)	3 (12)		14 (67)	5 (56)	1 (100)	2 (50)
Gender	Female	34 (56)	13 (50)		14 (67)	6 (67)	0 (0)	1 (25)
Ethnicity	White	51 (84)	19 (73)		19 (90)	8 (89)	1 (100)	4 (100)
	Non-White	10 (16)	7 (27)		2 (10)	1 (11)	0 (0)	0 (0)
Participant employment	Employed (FT/PT)	19 (31)	4 (15)		8 (38)	5 (56)	0 (0)	2 (50)
	Unemployed	17 (28)	8 (31)		3 (14)	4 (44)	0 (0)	2 (50)
	Retired	7 (11)	0 (0)		6 (29)	0 (0)	1 (100)	0 (0)
	N/A (child/HE)	18 (30)	14 (54)		4 (19)	0 (0)	0 (0)	0 (0)
Number of reviews, n		6 [4,7]	7 [4,8]		6 [3,6]	6 [5,7]	5 [5,5]	4 [2,5]
Period of reviews (years)		6 [3,7]	6 [3,7]		6 [2,6]	6 [5,6]	4 [4,4]	4 [2,6]

Results are presented as number (%) and median [IQR]

S=RDEB severe (RDEB-S), I=intermediate (RDEB-I), Inv=inversa (RDEB-Inv), PT=pretibial (RDEB-PT), Pru=pruriginosa (RDEB-Pru)

FT/PT=full time, part time

HE = in higher education

**Table 2:** Severity scores at index review (*n* = 61)

Variable	All RDEB^1^	RDEB-S	RDEB-I	RDEB-Inv	RDEB-Pru
n	**61**	**26**	**21**	**9**	**4**
iscorEB total score^2^	63 [41,82] (*n* = 56)	80 [62,102] (*n* = 23)	48 [28,67] (*n* = 19)	42 [37,59] (*n* = 9)	84 [58,97] (*n* = 4)
ISC clinician score^2^	21 [7,29] (*n* = 58)	28 [22,37] (*n* = 25)	8 [6,18] (*n* = 19)	6 [5,7] (*n* = 9)	22 [15,29] (*n* = 4)
ISP patient score^2^	43 [28,59] (*n* = 59)	48 [36,61] (*n* = 24)	28 [18,54] (*n* = 21)	36 [30,54] (*n* = 9)	57 [43,63] (*n* = 4)
BEBS total score^3^	27 [12,39] (*n* = 61)	40 [32,46] (*n* = 26)	15 [6,22] (*n* = 21)	9 [8,14] (*n* = 9)	26 [20,32] (*n* = 4)
Annual dressing time, hrs	332 [90,546] (*n* = 52)	520 [312,910] (*n* = 25)	87 [30,334] (*n* = 19)	61 [36,91] (*n* = 3)	377 [281,717] (*n* = 4)

Results are presented as median [IQR] (number)

S=RDEB severe (RDEB-S), I=intermediate (RDEB-I), Inv=inversa (RDEB-Inv), Pru=pruriginosa (RDEB-Pru)

^1^ One individual with pretibial RDEB was included in overall analysis but not separately reported

^2^ Instrument for scoring clinical outcomes of research for epidermolysis bullosa (iscorEB), maximum clinician (ISC) score of 138 and self-reported (ISP) score of 120

^3^ Birmingham Epidermolysis Bullosa Severity (BEBS) score, maximum of 100

### HRQoL for adults

The median QOLEB score at index review (*n* = 47) was 20 (13,29) out of a maximum 51, ranked, therefore, as severe. RDEB-Pru (*n* = 4) reported the worst HRQoL, 32 (26,35), although participant numbers were small, with RDEB-S (*n* = 26) scoring less highly at 26 (21,36). In contrast, RDEB-I (*n* = 18) and RDEB-Inv (*n* = 9) had the best HRQoL, respectively 16 (8,22) and 17 (13,21), ranked as moderate (Table 3). After adjusting for multiplicity, there were significant differences in subtype QOLEB scores between RDEB-S and RDEB-I (total score, *p*=.008, functioning subscore, *p*=.004) and RDEB-S and RDEB-Inv (total score, *p*=.020, functioning subscore, *p*=.015), although little variation in emotions subscores (Supplementary material 2).

**Table 3:** Quality of life scores (QOLEB^1^, PedsQL^2^) at index review (*n* = 61)

Variable	All RDEB^1^^, 3^	RDEB-S	RDEB-I	RDEB-Inv	RDEB-Pru
**QOLEB**, n	**47**	**15**	**18**	**9**	**4**
QOLEB score	20 [13,29] (*n* = 47)	26 [21,36] (*n* = 15)	16 [8,22] (*n* = 18)	17 [13,21] (*n* = 9)	32 [26,35] (*n* = 4)
QOLEB functioning subscore	15 [8,22] (*n* = 47)	22 [17,27] (*n* = 15)	11 [4,14] (*n* = 18)	11 [7,17] (*n* = 9)	24 [18,25] (*n* = 4)
QOLEB emotions subscore	5 [4,8] (*n* = 47)	5 [4,8] (*n* = 15)	5 [3,6] (*n* = 18)	6 [4,6] (*n* = 9)	8 [8,10] (*n* = 4)
**PedsQL**, n	**14**	**11**	**3**	**0**	**0**
PedsQL total score, parent	54 [42,56] (*n* = 14)	53 [43,55] (*n* = 11)	57 [47,57] (*n* = 3)		
PedsQL total score, patient	58 [51,60] (*n* = 13)	58 [49,63] (*n* = 11)	57 [56,58] (*n* = 2)		
PedsQL physical health, parent	34 [21,44] (*n* = 14)	34 [19,42] (*n* = 11)	44 [36,45] (*n* = 3)		
PedsQL physical health, patient	47 [38,56] (*n* = 13)	38 [31,59] (*n* = 11)	48 [48,49] (*n* = 2)		
PedsQL psychosocial health, parent	62 [53,66] (*n* = 14)	60 [53,68] (*n* = 11)	63 [53,63] (*n* = 3)		
PedsQL psychosocial health, patient	67 [60,70] (*n* = 13)	68 [61,71] (*n* = 11)	62 [59,64] (*n* = 2)		

Results are presented as median [IQR] (number)

S=RDEB severe (RDEB-S), I=intermediate (RDEB-I), Inv=inversa (RDEB-Inv), Pru=pruriginosa (RDEB-Pru)

^1^ One individual with pretibial RDEB was included in overall QOLEB analysis but not separately reported

^2^ Quality of Life in Epidermolysis Bullosa (QOLEB) maximum score of 51; includes subscores for functioning (maximum of 36) and emotions (maximum of 15); higher scores = worse HRQoL

^3^ Pediatric Quality of Life Inventory (PedsQL), total score maximum of 100; includes physical health summary score (“physical health”) maximum of 100; psychosocial health summary score (“psychosocial health”) maximum of 100; higher scores=better HRQoL

**Fig. 1:**
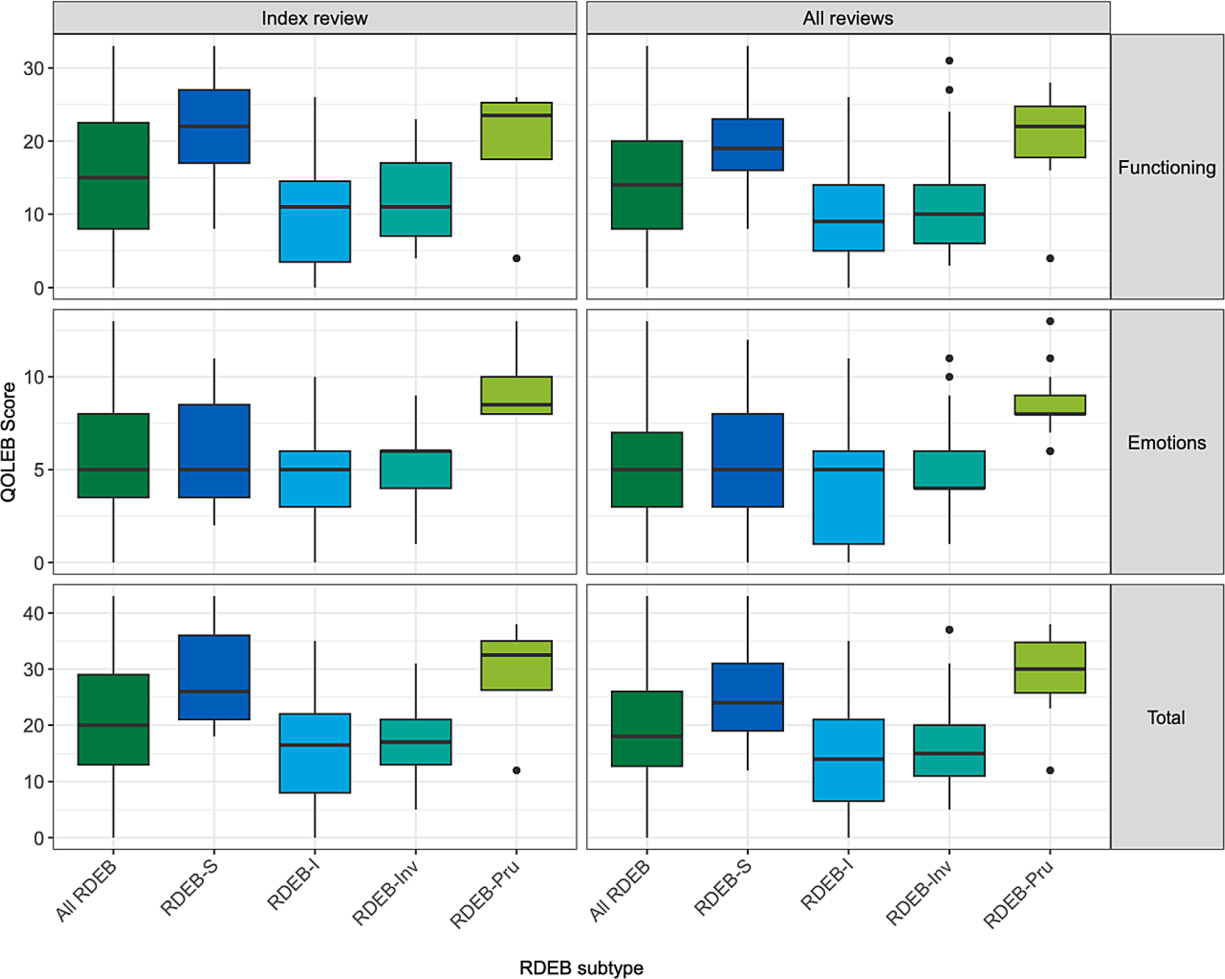
Box and whisker plot of Quality of Life in Epidermolysis Bullosa (QOLEB) at index review (*n* = 47) and all reviews (*n* = 244). QOLEB includes functioning and emotions subscores. Results are presented as median [IQR], with minimum and maximum data values, and outliers; data for 1 individual with pretibial RDEB are included only in “All RDEB”. Higher score indicates worse HRQoL. S=RDEB severe (RDEB-S), I=intermediate (RDEB-I), Inv=inversa (RDEB-Inv), Pru=pruriginosa (RDEB-Pru). These data are shown in tabular format in Supplementary material 3

These 47 adults reported a greater impact on functioning than emotions, respectively 15 (8,22) (42% of maximum functioning score of 36) and 5 (4,8) (33% of maximum emotions score of 15) (Fig. 1). Individuals with RDEB-Pru and RDEB-S reported the greatest impact on functioning, with RDEB-Pru also reporting the greatest impact on emotions (Table 3). Analysis of all QOLEB scores (*n* = 244) revealed similar findings to the index reviews (Fig. 1, Supplementary material 3), with a medium to large correlation between QOLEB functioning and emotions subscores for overall RDEB at index review and when considering all reviews, respectively *r* = .52 [0.28,0.70], *n* = 47 and *r* = .48 [0.37,0.57], *n* = 240 (Table 4a and 4b). Functioning and emotions subscores also correlated for subtypes RDEB-S, RDEB-I and RDEB-Inv when considering all reviews (Table 4b), but only for RDEB-I at index review (Table 4a). There was no correlation between subscores for RDEB-Pru.

**Table 4:** Correlations between HRQoL scores (QOLEB^1^, PedsQL^2^) and age and between functioning^1^/physical health^2^ and emotions^1^/psychosocial health^2^ subscores, (**a**) at index review (*n* = 61) and (**b**) when considering all reviews (*n* = 335)

a: Index reviews (*n* = 61)						
Variable1	Variable2	All RDEB^3^	RDEB-S	RDEB-I	RDEB-Inv	RDEB-Pru
**Index review**,** n**		**61**	**26**	**21**	**9**	**4**
QOLEB total score	Age (years)	*-0.43* *[-0.64*,*-0.16]* *(n* *= 47)*	**-0.58** **[-0.84**,**-0.09]** **(** ***n*** **= 15)**	-0.26 [-0.65,0.23] (*n* = 18)	-0.11 [-0.72,0.60] (*n* = 9)	-0.80 [-1.00,0.70] (*n* = 4)
QOLEB functioning subscore	Age (years)	*-0.48* *[-0.67*,*-0.22]* *(n* *= 47)*	**-0.59** **[-0.85**,**-0.12]** **(** ***n*** **= 15)**	-0.22 [-0.62,0.28] (*n* = 18)	-0.21 [-0.77,0.53] (*n* = 9)	-1.00 [-1.00,-1.00] (*n* = 4)
QOLEB emotions subscore	Age (years)	-0.20 [-0.46,0.10] (*n* = 47)	-0.23 [-0.67,0.32] (*n* = 15)	*-0.49* *[-0.78*,*-0.03]* *(n* *= 18)*	0.27 [-0.48,0.79] (*n* = 9)	-0.11 [-0.97,0.95] (*n* = 4)
PedsQL total score (parent)	Age (years)	-0.04 [-0.56,0.50] (*n* = 14)	0.06 [-0.56,0.64] (*n* = 11)	n/a (*n* = 3)	-	-
PedsQL Physical health (parent)	Age (years)	-0.35 [-0.74,0.22] (*n* = 14)	-0.35 [-0.78,0.33] (*n* = 11)	n/a (*n* = 3)	-	-
PedsQL Psycho-social health (parent)	Age (years)	0.46 [-0,10,0.80] (*n* = 14)	0.48 [-0,17,0.84] (*n* = 11)	n/a (*n* = 3)	-	-
PedsQL total score (patient)	Age (years)	0.02 [-0.54,0.57] (*n* = 13)	-0.01 [-0.61,0.59] (*n* = 11)	n/a (*n* = 2)	-	-
PedsQL Physical health (patient)	Age (years)	-0.14 [-0.64,0.44] (*n* = 13)	-0.20 [-0.72,0.45] (*n* = 11)	n/a (*n* = 2)	-	-
PedsQL Psycho-social health (patient)	Age (years)	0.42 [-0.17,0.71] (*n* = 13)	0.44 [-0.21,0.82] (*n* = 11)	n/a (*n* = 2)	-	-
QOLEB functioning subscore	QOLEB emotions subscore	**0.52** **[0.28**,**0.70]** **(** ***n*** **= 47)**	0.48 [-0.04,0.80] (*n* = 15)	**0.74** **[0.41**,**0.90]** **(** ***n*** **= 18)**	0.44 [-0.31,0.86] (*n* = 9)	0.11 [-0.95,0.97] (*n* = 4)
PedsQL physical health (parent)	PedsQL Psycho-social health (parent)	0.16 [-0.41,0.64] (*n* = 14)	0.11 [-0.53,0.67] (*n* = 11)	n/a (*n* = 3)	-	-
PedsQL physical health (patient)	PedsQL Psycho-social health (patient)	-0.21 [-0.68,0.39] (*n* = 13)	-0.27 [-0.75,0.40] (*n* = 11)	n/a (*n* = 2)	-	-

b: All reviews (*n* = 335)						
Variable1	Variable2	All RDEB^3^	RDEB-S	RDEB-I	RDEB-Inv	RDEB-Pru
**All reviews**,** n**		**335**	**166**	**99**	**51**	**14**
QOLEB total score	Age (years)	*-0.30* *[-0.41*,*-0.18]* *(n* *= 240)*	0.02 [-0.20,0.24] (*n* = 81)	-0.11 [-0.31,0.10] (*n* = 91)	*-0.39* *[-0.61*,*-0.13]* *(n* *= 49)*	-0.40 [-0.77,0.16] (*n* = 14)
QOLEB functioning subscore	Age (years)	*-0.36* *[-0.47*,*-0.25]* *(n* *= 241)*	-0.01 [-0.23,0.21] (*n* = 81)	-0.11 [-0.31,0.10] (*n* = 92)	*-0.42* *[-0.63*,*-0.16]* *(n* *= 49)*	-0.39 [-0.76,0.18] (*n* = 14)
QOLEB emotions subscore	Age (years)	-0.10 [-0.23,0.02] (*n* = 244)	0.07 [-0.15,0.29] (*n* = 81)	-0.11 [-0.31,0.09] (*n* = 93)	-0.19 [-0.44,0.09] (*n* = 51)	-0.21 [-0.67,0.36] (*n* = 14)
PedsQL total score (parent)	Age (years)	-0.06 [-0.26,0.15] (*n* = 88)	0.01 [-0.21,0.22] (*n* = 83)	-0.80 [-0.99,0.28] (*n* = 5)	-	-
PedsQL Physical health (parent)	Age (years)	*-0.30 * *[-0.48*,*-0.10] (n = 89)*	-0.27 [-0.46,-0.06] (*n* = 84)	-0.80 [-0.99,0.28] (*n* = 5)	-	-
PedsQL Psycho-social health (parent)	Age (years)	-0.20 [-0,39,0.01] (*n* = 88)	-0.20 [-0,40,0.01] (*n* = 83)	-0.10 [-0.86,0.90] (*n* = 5)	-	-
PedsQL total score (patient)	Age (years)	-0.04 [-0.28,0.21] (*n* = 63)	-0.06 [-0.31,0.19] (*n* = 61)	n/a (*n* = 2)	-	-
PedsQL Physical health (patient)	Age (years)	-0.12 [-0.35,0.13] (*n* = 64)	-0.14 [-0.37,0.12] (*n* = 62)	n/a (*n* = 2)	-	-
PedsQL Psycho-social health (patient)	Age (years)	0.04 [-0.21,0.29] (*n* = 63)	0.01 [-0.24,0.26] (*n* = 61)	n/a (*n* = 2)	-	-
QOLEB functioning subscore	QOLEB emotions subscore	*0.48* *[0.37*,*0.57]* *(n* *= 240)*	*0.36* *[0.15*,*0.53]* *(n* *= 81)*	**0.67** **[0.53**,**0.77]** **(** ***n*** **= 91)**	*0.41* *[0.15*,*0.62]* *(n* *= 49)*	0.12 [-0.44,0.61] (*n* = 14)
PedsQL physical health (parent)	PedsQL Psycho-social health (parent)	**0.60** **[0.44**,**0.72]** **(** ***n*** **= 88)**	**0.59** **[0.43**,**0.71]** **(** ***n*** **= 83)**	0.40 [-0.75,0.95] (*n* = 5)	-	-
PedsQL physical health (patient)	PedsQL Psycho-social health (patient)	*0.30* *[0.06*,*0.51]* *(n* *= 63)*	*0.31* *[0.07*,*0.52]* *(n* *= 61)*	n/a (*n* = 2)	-	-

Results are presented as correlation [95% CI] (number), calculated using Spearman’s rank correlation

S=RDEB severe (RDEB-S), I=intermediate (RDEB-I), Inv=inversa (RDEB-Inv), Pru=pruriginosa (RDEB-Pru)

Associations are significant (*p*<.05) if the 95% CI does not contain 0

Correlations are interpreted as **large**, ***r*=.50 − 1.0**; *medium*, *r*=.*30*-.*49*; small, *r*=.10-.29. (Cohen, 1988)

Correlations where *n* < 10 should be considered with caution as the associations could be spurious

^1^ Quality of Life in Epidermolysis Bullosa (QOLEB) includes subscores for functioning and emotions; higher scores = worse HRQoL; Negative QOLEB correlations indicate QOL scores are worse when younger and improve with age

^2^ Pediatric Quality of Life Inventory (PedsQL) includes physical health summary score (“physical health”) and psychosocial health summary score (“psychosocial health”); higher score=better HRQoL; Negative PedsQL correlations indicate QOL scores are better when younger and worsen with age

^3^ Reviews by one individual with pretibial RDEB (*n* = 5) were included in overall QOLEB analysis but not separately reported

QOLEB total scores and functioning subscores were negatively correlated with age at both index review (respectively − 0.43 [-0.64,-0.16] and − 0.48 [-0.67,-0.22]) and when considering all 240 reviews (respectively − 0.30 [-0.41,-0.18] and − 0.36 [-0.47,-0.25]), with worse HRQoL reported by younger participants (Table 4a and b). However, correlations between these scores for RDEB subtypes were only significant for RDEB-S at index review (respectively, -0.58 [-0.84,-0.09] and − 0.59 [-0.85,-0.12], *n* = 15) (Table 4a) and for RDEB-Inv when considering all reviews (respectively, -0.39 [-0.61,-0.13] and − 0.42 [-0.63,-0.16], *n* = 49) (Table 4b). QOLEB functioning and total scores also had a large positive correlation with severity scores (iscorEB and BEBS) at index review and when considering all reviews, indicating worse HRQoL reported by those with more severe symptoms (Table 5a and 5b). However, emotions subscores had a large correlation only with self-reported severity scores (ISP), not with iscorEB clinician scores (ISC), for all RDEB and subtypes (except RDEB-Pru) (Table 5a and 5b). There was also a medium correlation (index review, Table 5a) and small correlation (all reviews) with BEBS scores for all RDEB, although not for the individual subtypes except for RDEB-I when considering all reviews (Table 5b).

**Table 5:** Correlations between QOLEB^1^ scores and EB severity scores (iscorEB^2^, BEBS^3^) at (**a**) index reviews (*n* = 47) and (**b**) when considering all reviews (*n* = 245)

a: index reviews (*n* = 47)						
Variable 1	Variable 2	All RDEB^4^	RDEB-S	RDEB-I	RDEB-Inv	RDEB-Pru
**Index review**,** n**		**47**	**15**	**18**	**9**	**4**
QOLEB total score	ISC, clinician score	**0.63** **[0.41**,**0.78]** **(** ***n*** **= 46)**	0.43 [-0.10,0.77] (*n* = 15)	0.37 [-0.13,0.72] (*n* = 17)	0.14 [-0.58,0.74] (*n* = 9)	0.80 [-0.70,1.00] (*n* = 4)
QOLEB total score	ISP, patient score	**0.91** **[0.85**,**0.95]** **(** ***n*** **= 46)**	**0.77** **[0.40**,**0.92]** **(** ***n*** **= 14)**	**0.92** **[0.79**,**0.97]** **(** ***n*** **= 18)**	0.87 [0.47,0.97] (*n* = 9)	1.00 [1.00,1.00] (*n* = 4)
QOLEB total score	iscorEB total score	**0.89** **[0.81**,**0.94]** **(** ***n*** **= 45)**	**0.72** **[0.30**,**0.90]** **(** ***n*** **= 14)**	**0.88** **[0.70**,**0.96]** **(** ***n*** **= 17)**	0.92 [0.67,0.98] (*n* = 9)	1.00 [1.00,1.00] (*n* = 4)
QOLEB total score	BEBS total score	**0.69** **[0.50**,**0.81]** **(** ***n*** **= 47)**	0.19 [-0.35,0.64] (*n* = 15)	**0.64** **[0.24**,**0.85]** **(** ***n*** **= 18)**	0.44 [-0.32,0.85] (*n* = 9)	0.80 [-0.70,1.00] (*n* = 4)
QOLEB functioning score	ISC, clinician score	**0.65** **[0.44**,**0.79]** **(** ***n*** **= 46)**	0.46 [-0.07,0.79] (*n* = 15)	0.35 [-0.16,0.71] (*n* = 17)	0.18 [-0.55,0.75] (*n* = 9)	1.00 [1.00,1.00] (*n* = 4)
QOLEB functioning score	ISP, patient score	**0.87** **[0.78**,**0.93]** **(** ***n*** **= 46)**	**0.69** **[0.26**,**0.89]** **(** ***n*** **= 14)**	**0.92** **[0.80**,**0.97]** **(** ***n*** **= 18)**	0.86 [0.46,0.97] (*n* = 9)	0.80 [-0.70,1.00] (*n* = 4)
QOLEB functioning score	iscorEB total score	**0.87** **[0.77**,**0.93]** **(** ***n*** **= 45)**	**0.67** **[0.21**,**0.88]** **(** ***n*** **= 14)**	**0.88** **[0.69**,**0.96]** **(** ***n*** **= 17)**	0.93 [0.69,0.99] (*n* = 9)	0.80 [-0.70,1.00] (*n* = 4)
QOLEB functioning score	BEBS total score	**0.71** **[0.54**,**0.83]** **(** ***n*** **= 47)**	0.18 [-0.36,0.64] (*n* = 15)	**0.66** **[0.28**,**0.86]** **(** ***n*** **= 18)**	0.49 [-0.25,0.87] (*n* = 9)	1.00 [1.00,1.00] (*n* = 4)
QOLEB emotions score	ISC, clinician score	0.28 [-0.01,0.53] (*n* = 46)	0.24 [-0.31,0.67] (*n* = 15)	0.40 [-0.09,0.74] (*n* = 17)	0.19 [-0.55,0.76] (*n* = 9)	0.11 [-0.95,0.97] (*n* = 4)
QOLEB emotions score	ISP, patient score	**0.64** **[0.43**,**0.78]** **(** ***n*** **= 46)**	**0.75** **[0.37**,**0.92]** **(** ***n*** **= 14)**	**0.70** **[0.34**,**0.88]** **(** ***n*** **= 18)**	0.56 [-0.17,0.89] (*n* = 9)	0.63 [-0.84,0.99] (*n* = 4)
QOLEB emotions score	iscorEB total score	**0.53** **[0.29**,**0.72]** **(** ***n*** **= 45)**	**0.64** **[0.16**,**0.87]** **(** ***n*** **= 14)**	**0.68** **[0.30**,**0.88]** **(** ***n*** **= 17)**	0.49 [-0.26,0.87] (*n* = 9)	0.63 [-0.84,0.99] (*n* = 4)
QOLEB emotions score	BEBS total score	*0.33* *[0.05*,*0.57]* *(n* *= 47)*	0.17 [-0.37,0.63] (*n* = 15)	0.44 [-0.03,0.75] (*n* = 18)	0.10 [-0.60,0.72] (*n* = 9)	0.11 [-0.95,0.97] (*n* = 4)

b: when considering all reviews (*n* = 245)						
Variable 1	Variable 2	All RDEB^4^	RDEB-S	RDEB-I	RDEB-Inv	RDEB-Pru
**All reviews**,** n**		**245**	**81**	**94**	**51**	**14**
QOLEB total score	ISC, clinician score	**0.58** **[0.48**,**0.67]** **(** ***n*** **= 179)**	*0.41* *[0.20*,*0.59]* *(n* *= 72)*	0.29 [0.04,0.51] (*n* = 59)	**0.52** **[0.23**,**0.73]** **(** ***n*** **= 35)**	0.40 [-0.22,0.79] (*n* = 12)
QOLEB total score	ISP, patient score	**0.83** **[0.78**,**0.86]** **(** ***n*** **= 233)**	**0.79** **[0.69**,**0.86]** **(** ***n*** **= 77)**	**0.85** **[0.78**,**0.90]** **(** ***n*** **= 90)**	**0.85** **[0.74**,**0.91]** **(** ***n*** **= 48)**	0.36 [-0.24,0.76] (*n* = 13)
QOLEB total score	iscorEB total score	**0.86** **[0.81**,**0.89]** **(** ***n*** **= 176)**	**0.78** **[0.67**,**0.86]** **(** ***n*** **= 69)**	**0.80** **[0.69**,**0.88]** **(** ***n*** **= 59)**	**0.87** **[0.76**,**0.94]** **(** ***n*** **= 35)**	0.50 [-0.10,0.83] (*n* = 12)
QOLEB total score	BEBS total score	**0.70** **[0.62**,**0.76]** **(** ***n*** **= 224)**	0.26 [0.03,0.46] (*n* = 74)	**0.67** **[0.54**,**0.78]** **(** ***n*** **= 85)**	*0.47* *[0.21*,*0.67]* *(n* *= 47)*	**0.68** **[0.21**,**0.90]** **(** ***n*** **= 13)**
QOLEB functioning score	ISC, clinician score	**0.65** **[0.56**,**0.73]** **(** ***n*** **= 180)**	**0.53** **[0.34**,**0.68]** **(** ***n*** **= 72)**	*0.31* *[0.07*,*0.53]* *(n* *= 60)*	**0.56** **[0.28**,**0.75]** **(** ***n*** **= 35)**	**0.66** **[0.15**,**0.90]** **(** ***n*** **= 12)**
QOLEB functioning score	ISP, patient score	**0.78** **[0.72**,**0.83]** **(** ***n*** **= 233)**	**0.68** **[0.54**,**0.79]** **(** ***n*** **= 77)**	**0.85** **[0.78**,**0.90]** **(** ***n*** **= 90)**	**0.80** **[0.66**,**0.88]** **(** ***n*** **= 48)**	0.16 [-0.43,0.66] (*n* = 13)
QOLEB functioning score	iscorEB total score	**0.84** **[0.80**,**0.88]** **(** ***n*** **= 176)**	**0.78** **[0.66**,**0.86]** **(** ***n*** **= 69)**	**0.78** **[0.65**,**0.86]** **(** ***n*** **= 59)**	**0.86** **[0.73**,**0.93]** **(** ***n*** **= 35)**	0.51 [-0.09,0.84] (*n* = 12)
QOLEB functioning score	BEBS total score	**0.75** **[0.68**,**0.80]** **(** ***n*** **= 225)**	*0.34* *[0.13*,*0.53]* *(* ***n*** **= 74)**	**0.70** **[0.58**,**0.80]** **(** ***n*** **= 86)**	**0.51** **[0.26**,**0.70]** **(** ***n*** **= 47)**	**0.70** **[0.23**,**0.90]** **(** ***n*** **= 13)**
QOLEB emotions score	ISC, clinician score	0.11 [-0.04,0.25] (*n* = 183)	-0.03 [-0.26,0.20] (*n* = 72)	0.22 [-0.04,0.44] (*n* = 61)	0.13 [-0.21,0.43] (*n* = 37)	-0.54 [-0.85,0.05] (*n* = 12)
QOLEB emotions score	ISP, patient score	**0.62** **[0.53**,**0.69]** **(** ***n*** **= 237)**	**0.66** **[0.51**,**0.77]** **(** ***n*** **= 77)**	**0.65** **[0.51**,**0.75]** **(** ***n*** **= 92)**	**0.60** **[0.38**,**0.75]** **(** ***n*** **= 50)**	0.20 [-0.40,0.67] (*n* = 13)
QOLEB emotions score	iscorEB total score	*0.44* *[0.32*,*0.55]* *(n* *= 180)*	*0.43* *[0.21*,*0.60]* *(n* *= 69)*	**0.63** **[0.44**,**0.76]** **(** ***n*** **= 61)**	*0.46* *[0.17*,*0.69]* *(n* *= 37)*	-0.15 [-0.67,0.46] (*n* = 12)
QOLEB emotions score	BEBS total score	0.27 [0.15,0.39] (*n* = 228)	-0.05 [-0.28,0.18] (*n* = 74)	*0.47* *[0.29*,*0.62]* *(n* *= 87)*	0.06 [-0.23,0.33] (*n* = 49)	0.09 [-0.49,0.61] (*n* = 13)

Results are presented as correlation [95% CI] (number), calculated using Spearman’s rank correlation

S=RDEB severe (RDEB-S), I=intermediate (RDEB-I), Inv=inversa (RDEB-Inv), Pru=pruriginosa (RDEB-Pru)

Associations are significant (*p*<.05) if the 95% CI does not contain 0

Correlations are interpreted as **large**, ***r***=**.50 − 1.0**; *medium*, *r*=.*30*-.*49*; small, *r*=.10-.29. (Cohen, 1988)

Correlations where *n* < 10 should be considered with caution as the associations could be spurious, so are not highlighted in the table

^1^ Quality of Life in Epidermolysis Bullosa (QOLEB) includes functioning and emotions subscores; higher scores = worse HRQoL

^2^ Instrument for Scoring Clinical Outcomes for Epidermolysis Bullosa (iscorEB); includes clinician score (ISC) and patient score (ISP); higher scores = worse severity

^3^ Birmingham Epidermolysis Bullosa Severity score (BEBS); higher scores = worse severity

^4^ Reviews by one individual with pretibial RDEB (*n* = 5) were included in overall analysis but not separately reported

**Fig. 2:**
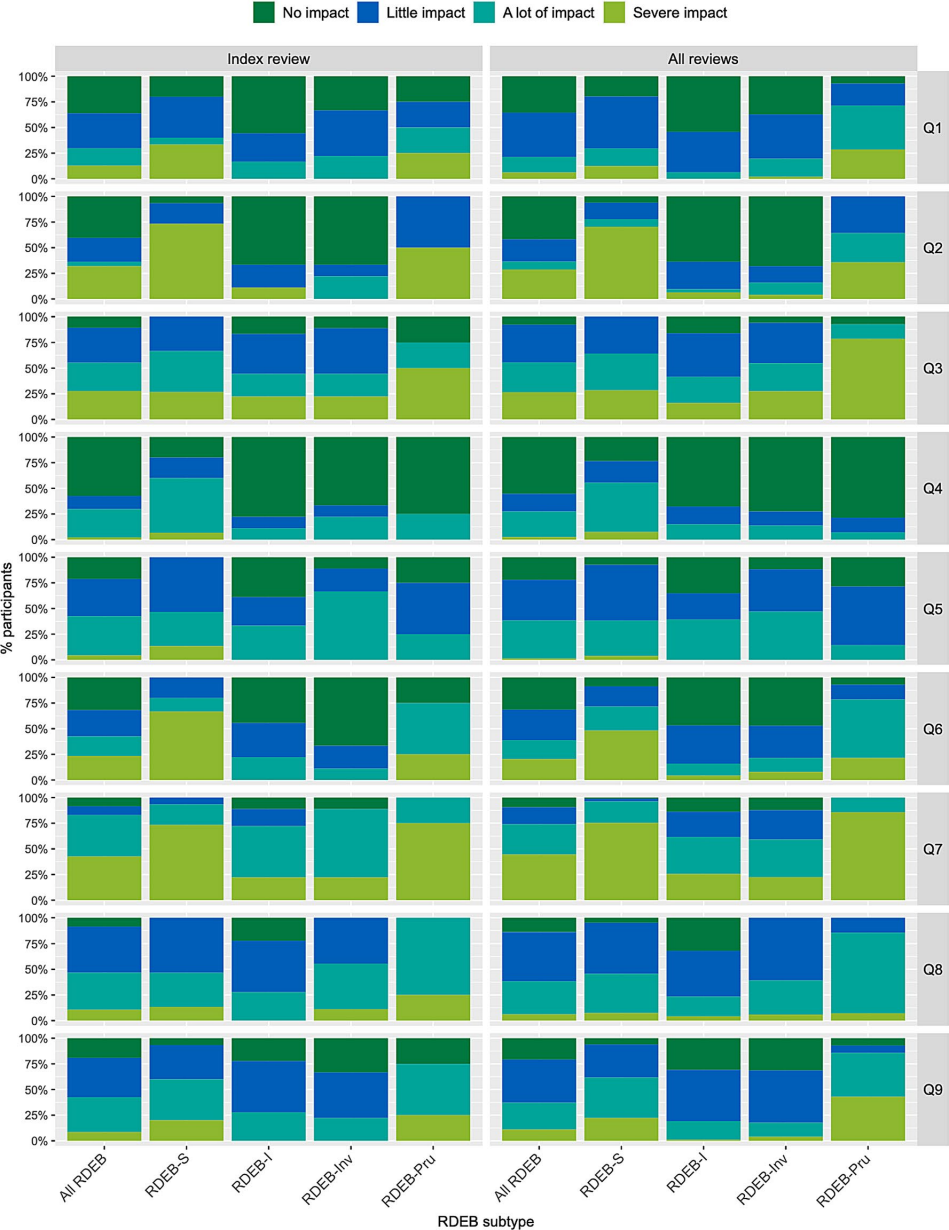

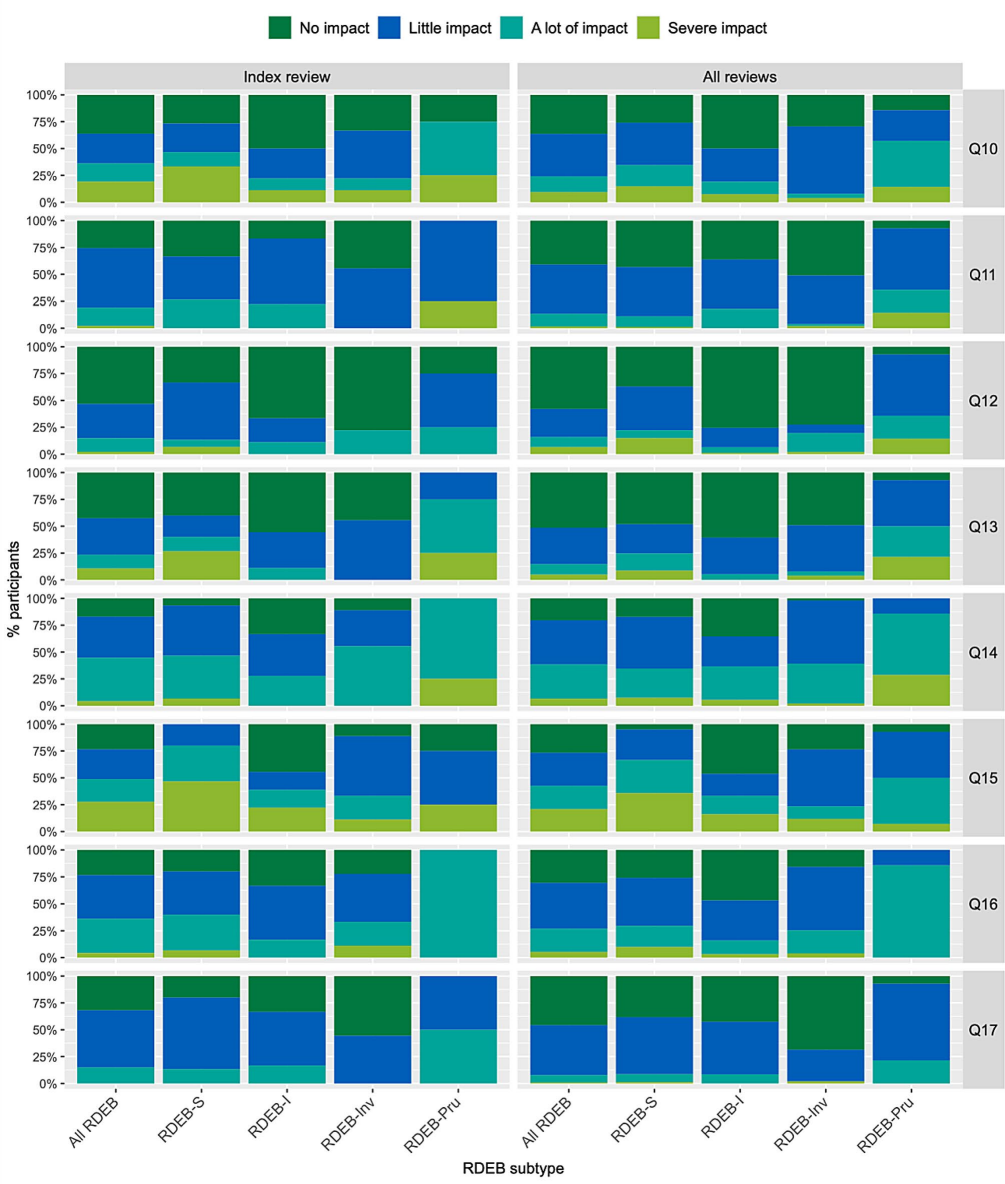
Quality of Life in Epidermolysis Bullosa (QOLEB), distribution of responses at index review (*n* = 47) and all reviews (*n* = 244). Results are presented as ordinal level data; data for 1 individual with pretibial RDEB are included only in “All RDEB”. Q1: Movement at home, Q2: Bathing, Q3: Pain, Q4: Writing, Q5: Eating, Q6: Shopping, Q7: Sports, Q8: Frustration, Q9: Movement outside, Q10: Family relationships, Q11: Embarrassment, Q12: Home modifications, Q13: Friendships, Q14: Anxiety, Q15: Financial impact, Q16: Depression, Q17: Uncomfortable, S=RDEB severe (RDEB-S), I=intermediate (RDEB-I), Inv=inversa (RDEB-Inv), Pru=pruriginosa (RDEB-Pru), These data are shown in tabular format in Supplementary materials 4 and 5

Figure 2 shows the distribution of responses to each of the 17 QOLEB items, for all RDEB and by subtype, with similar findings when considering index reviews (*n* = 47) or all available reviews (*n* = 245); supplementary materials 4 and 5 report these data in tabular format. At index review, most participants with RDEB-S required assistance ‘most’ or ‘all’ of the time for bathing/showering (Q2: 73%) or going shopping (Q6: 80%), had ‘frequent’ or ‘constant’ pain (Q3: 67%), difficulty writing (Q4: 80%) and experienced ‘a lot’ or ‘severe’ restriction in movement outside their home (Q9: 60%); other RDEB subtypes reported little or no impact for these issues, except that three out of four individuals with RDEB-Pru reported ‘frequent’ or ‘constant’ pain (Q3) and ‘a lot’ or ‘severe’ restriction in movement outside their home (Q9). All subtypes reported the need to avoid some or all sports (Q7: 83%), although those with RDEB-S were more likely to avoid all sports (73%, compared with < 25% for RDEB-I or RDEB-Inv). Most individuals with RDEB-S reported ‘great’ or ‘severe’ financial impact (Q15: 80%) whereas other subtypes mostly reported ‘no’ or ‘slight’ impact (Q15: 61–75%). Most participants in all subtypes reported ‘little’ or ‘no’ for the following: embarrassment (Q11; 81%), feeling uncomfortable due to teasing (Q17; 85%), feeling depressed (Q16; 63%), impact on family relationships (Q10; 64%), impact on friendships (Q13; 77%) frustration (Q8; 54%); the exception was all four individuals with RDEB-Pru who reported feeling depressed ‘a lot’. Few reported needing to undertake a lot or extensive modifications of their home (Q12: 15%). Nearly half of all subtypes felt ‘a lot’ or ‘extremely’ anxious because of their EB (Q14: 44%), with individuals with RDEB-Pru reporting the greatest anxiety (100%) and those with RDEB-I the least (28%). However, 54% of RDEB-S and 44% of RDEB-Inv reported little/no anxiety. A similar pattern is seen when considering all reviews, with a few exceptions such as greater numbers with RDEB-S (65%) and RDEB-Inv (61%) who reported little/no anxiety (Q14) and 13 of the 14 RDEB-Pru reviews (93%) reported frequent/constant pain (Q3).

### HRQoL for children

PedsQL scores were available for 14 child participants, 11 with RDEB-S and 3 with RDEB-I; one child with RDEB-I aged 2–4 years had only parent responses.

**Fig. 3:**
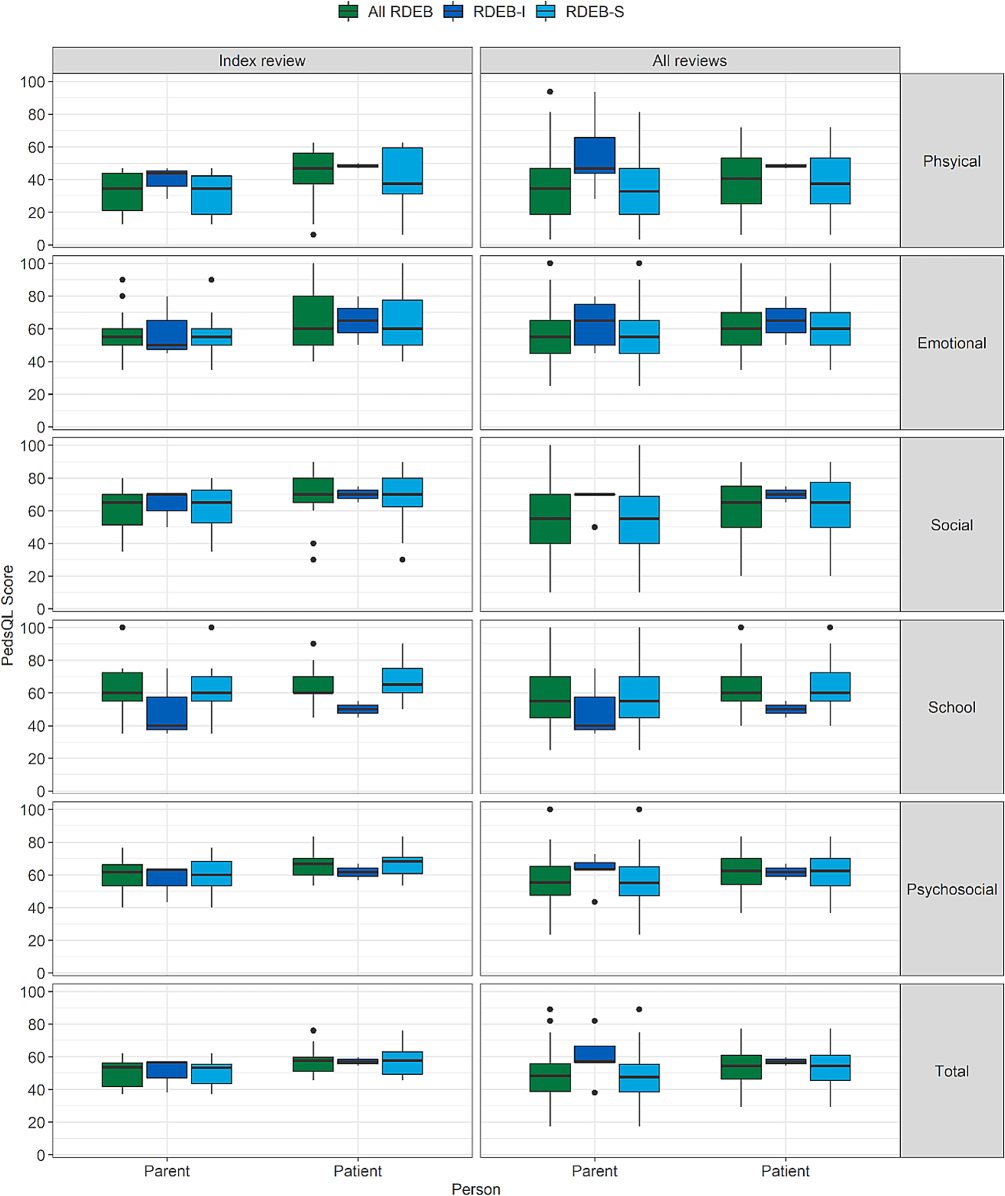
Box and whisker plot of Pediatric Quality of Life Inventory (PedsQL) at index review (*n* = 14) and all reviews (*n* = 89). PedsQL comprises 4 domains: physical domain, which is the physical subscore, and emotional, social and school functioning domains which together comprise the psychosocial subscore. Results are presented as median [IQR], with minimum and maximum data values, and outliers. Higher scores indicate better HRQoL. S=RDEB severe (RDEB-S), I=intermediate (RDEB-I). These data are shown in tabular format in Supplementary material 6

Children with RDEB-S and their parents reported worse physical health (lower scores) than psychosocial health at index review and when considering all reviews (Fig. 3; Table 3, Supplementary material 6); there were too few child participants to be confident about PedsQL findings at index review (*n* = 14) including correlations between PedsQL and other scores (Table 6a). Investigation of the relationship between HRQoL and symptom severity in all reviews (*n* = 77) revealed worse HRQoL associated with worse severity scores although there were differences between parent (-0.51 [-0.66,-0.32]) and patient/child perception (-0.70 [-0.82,-0.54]) of HRQoL (Table 6b). Child PedsQL scores had a large negative correlation with ISC, ISP, and therefore iscorEB (-0.68 [-0.83,-0.43]), and a medium negative correlation between PedsQL and BEBS (-0.30 [-0.52,-0.04]), indicating greater symptom severity was associated with worse HRQoL. However, parent PedsQL scores had moderate negative correlations with iscorEB (-0.43 [-0.65,-0.14]) and small negative correlations for the clinician scores (ISC, BEBS), respectively, -0.29 [-0.53,-0.02] and − 0.25 [-0.45,-0.03], respectively suggesting less or no association of their HRQoL scores with their child’s symptom severity.

**Table 6:** Correlations between PedsQL^1^ (parent and child) scores and EB severity scores (iscorEB^2^, BEBS^3^) at (a) index reviews (*n* = 14) and (b) when considering all reviews (*n* = 89)

a: index reviews (*n* = 14)										
Variable 1	Variable 2		All RDEB Parent^4^		RDEB-S Parent		All RDEB Child^4^		RDEB-S Child	
Index review, n			**14**		**11**		**13**		**11**	
PedsQL total score	ISC, clinician score		-0.08 [-0.62,0.52] (*n* = 12)		-0.26 [-0.77,0.44] (*n* = 10)		-0.56 [-0.86,0.01] (*n* = 12)		**-0.71** **[-0.93**,**-0.15]** **(** ***n*** **= 10)**	
PedsQL total score	ISP, patient score		-0.33 [-0.75,0.27] (*n* = 13)		-0.28 [-0.77,0.42] (*n* = 10)		**-0.78** **[-0.93**,**-0.37]** **(** ***n*** **= 12)**		**-0.89** **[-0.97**,**-0.60]** **(** ***n*** **= 10)**	
PedsQL total score	iscorEB total score		-0.14 [-0.68,0.50] (*n* = 11)		-0.19 [-0.76,0.54] (*n* = 9)		**-0.71** **[-0.92**,**-0.19]** **(** ***n*** **= 11)**		-0.77 [-0.95,-0.22] (*n* = 9)	
PedsQL total score	BEBS total score		-0.11 [-0.60,0.45] (*n* = 14)		-0.17 [-0.70,0.48] (*n* = 11)		-0.30 [-0.73,0.30] (*n* = 13)		-0.42 [-0.81,0.25] (*n* = 11)	
PedsQL physical health	ISC, clinician score		-0.49 [-0.83,0.12] (*n* = 12)		**-0.63** **[-0.90**,**-0.01]** **(** ***n*** **= 10)**		**-0.70** **[-0.91**,**-0.21]** **(** ***n*** **= 12)**		**-0.76** **[-0.94**,**-0.26]** **(** ***n*** **= 10)**	
PedsQL physical health	ISP, patient score		-0.54 [-0.84,0.02] (*n* = 13)		-0.54 [-0.87,0.13] (*n* = 10)		**-0.79** **[-0.94**,**-0.41]** **(** ***n*** **= 12)**		**-0.84** **[-0.96**,**-0.44]** **(** ***n*** **= 10)**	
PedsQL physical health	iscorEB total score		-0.45 [-0.83,0.20] (*n* = 11)		-0.44 [-0.85,0.32] (*n* = 9)		**-0.80** **[-0.94**,**-0.38]** **(** ***n*** **= 11)**		-0.78 [-0.95,-0.23] (*n* = 9)	
PedsQL physical health	BEBS total score		-0.37 [-0.75,0.19] (*n* = 14)		-0.38 [-0.80,0.28] (*n* = 11)		-0.49 [-0.82,0.08] (*n* = 13)		-0.52 [-0.85,0.12] (*n* = 11)	
PedsQL psycho-social health	ISC, clinician score		0.15 [-0.46,0.67] (*n* = 12)		0.00 [-0.63,0.63] (*n* = 10)		-0.12 [-0.65,0.49] (*n* = 12)		-0.33 [-0.80,0.37] (*n* = 10)	
PedsQL psycho-social health	ISP, patient score		-0.08 [-0.61,0.49] (*n* = 13)		-0.02 [-0.64,0.62] (*n* = 10)		-0.52 [-0.84,0.07] (*n* = 12)		-0.50 [-0.86,0.18] (*n* = 10)	
PedsQL psycho-social health	iscorEB total score		0.04 [-0.58,0.62] (*n* = 11)		0.00 [-0.66,0.66] (*n* = 9)		-0.42 [-0.81,0.25] (*n* = 11)		-0.44 [-0.85,0.32] (*n* = 9)	
PedsQL psycho-social health	BEBS total score		0.08 [-0.47,0.59] (*n* = 14)		0.00 [-0.60,0.60] (*n* = 11)		-0.02 [-0.57,0.53] (*n* = 13)		-0.24 [-0.73,0.42] (*n* = 11)	

b: Considering all reviews (*n* = 89)										
Variable 1	Variable 2	All RDEB Parent		RDEB-S Parent		RDEB-I Parent		All RDEB Child^4^		RDEB-S Child
Index review, n		**89**		**84**		**5**		**55**		**2**
PedsQL total score	ISC, clinician score	-0.29 [-0.53,-0.02] (*n* = 51)		*-0.33* *[-0.56*,*-0.05]* *(n* *= 49)*		n/a (*n* = 2)		**-0.58** **[-0.76**,**-0.33]** **(** ***n*** **= 39)**		**-0.62** **[-0.79**,**-0.37]** **(** ***n*** **= 37)**
PedsQL total score	ISP, patient score	**-0.51** **[-0.66**,**-0.32]** **(** ***n*** **= 77)**		*-0.48* *[-0.64*,*-0.27]* *(n* *= 72)*		-0.60 [-0.97,0.60] (*n* = 5)		**-0.70** **[-0.82**,**-0.54]** **(** ***n*** **= 55)**		**-0.72** **[-0.83**,**-0.55]** **(** ***n*** **= 53)**
PedsQL total score	iscorEB total score	*-0.43* *[-0.65*,*-0.14]* *(n* *= 42)*		*-0.47* *[-0.68*,*-0.18]* *(n* *= 40)*		n/a (*n* = 2)		**-0.68** **[-0.83**,**-0.43]** **(** ***n*** **= 32)**		**-0.71** **[-0.85**,**-0.47]** **(** ***n*** **= 30)**
PedsQL total score	BEBS total score	-0.25 [-0.45,-0.03] (*n* = 78)		-0.19 [-0.41,0.04] (*n* = 73)		-0.72 [-0.98,0.45] (*n* = 5)		*-0.30* *[-0.52*,*-0.04]* *(n* *= 55)*		*-0.31* *[-0.54*,*-0.05]* *(n* *= 53)*
PedsQL physical health	ISC, clinician score	*-0.49* *[-0.67*,*-0.25]* *(n* *= 52)*		**-0.52** **[-0.70**,**-0.28]** **(** ***n*** **= 50)**		n/a (*n* = 2)		*-0.45* *[-0.67*,*-0.16]* *(n* *= 39)*		*-0.46* *[-0.68*,*-0.16]* *(n* *= 37)*
PedsQL physical health	ISP, patient score	**-0.50** **[-0.65**,**-0.32]** **(** ***n*** **= 78)**		*-0.47* *[-0.63*,*-0.27]* *(n* *= 73)*		-0.60 [-0.97,0.60] (*n* = 5)		**-0.53** **[-0.70**,**-0.32]** **(** ***n*** **= 56)**		**-0.55** **[-0.71**,**-0.33]** **(** ***n*** **= 54)**
PedsQL physical health	iscorEB total score	**-0.51** **[-0.70**,**-0.25]** **(** ***n*** **= 43)**		**-0.53** **[-0.72**,**-0.27]** **(** ***n*** **= 41)**		n/a (*n* = 2)		**-0.54** **[-0.75**,**-0.23]** **(** ***n*** **= 32)**		**-0.53** **[-0.75**,**-0.21]** **(** ***n*** **= 30)**
PedsQL physical health	BEBS total score	*-0.48* *[-0.63*,*-0.29]* *(n* *= 79)*		*-0.44* *[-0.61*,*-0.23]* *(n* *= 74)*		-0.72 [-0.98,0.45] (*n* = 5)		-0.29 [-0.52,-0.03] (*n* = 55)		-0.29 [-0.52,-0.02] (*n* = 53)
PedsQL psycho-social health	ISC, clinician score	-0.17 [-0.43,0.11] (*n* = 51)		-0.20 [-0.46,0.08] (*n* = 49)		n/a (*n* = 2)		**-0.52** **[-0.72**,**-0.24]** **(** ***n*** **= 39)**		**-0.57** **[-0.76**,**-0.31]** **(** ***n*** **= 37)**
PedsQL psycho-social health	ISP, patient score	*-0.40* *[-0.57*,*-0.19]* *(n* *= 77)*		*-0.36* *[-0.55*,*-0.14]* *(n* *= 72)*		-0.60 [-0.97,0.60] (*n* = 5)		**-0.62** **[-0.76**,**-0.42]** **(** ***n*** **= 55)**		**-0.62** **[-0.76**,**-0.42]** **(** ***n*** **= 53)**
PedsQL psycho-social health	iscorEB total score	*-0.34* *[-0.58*,*-0.04]* *(n* *= 42)*		*-0.37* *[-0.61*,*-0.07]* *(n* *= 40)*		n/a (*n* = 2)		**-0.65** **[-0.81**,**-0.39]** **(** ***n*** **= 32)**		**-0.69** **[-0.84**,**-0.44]** **(** ***n*** **= 30)**
PedsQL psycho-social health	BEBS total score	-0.12 [-0.34,0.10] (*n* = 78)		-0.07 [-0.29,0.16] (*n* = 73)		-0.72 [-0.98,0.45] (*n* = 5)		-0.23 [-0.47,0.04] (*n* = 55)		-0.26 [-0.49,0.02] (*n* = 53)

Results are presented as correlation [95% CI] (number), calculated using Spearman’s rank correlation

S=RDEB severe (RDEB-S), I=intermediate (RDEB-I)

Associations are significant (*p*<.05) if the 95% CI does not contain 0

Correlations are interpreted as **large**, ***r***=**.50 − 1.0**; *medium*, *r*=.*30*-.*49*; small, *r*=.10-0.29. (Cohen, 1988)

Correlations where *n* < 10 should be considered with caution as the associations could be spurious, so are not highlighted in the table

^1^ Pediatric Quality of Life Inventory (PedsQL) includes physical health summary score (“physical health”) and psychosocial health summary score (“psychosocial health”); higher score=better HRQoL

^2^ Instrument for Scoring Clinical Outcomes for Epidermolysis Bullosa (iscorEB); includes clinician score (ISC) and patient score (ISP); higher score=worse severity

^3^ Birmingham Epidermolysis Bullosa Severity score (BEBS); higher score=worse severity

^4^ Reviews by those with RDEB-I (3 parents and 2 children) were included in overall analysis but not separately reported

When considering all reviews, children had more difficulty running and playing sports than walking (Supplementary material 6). Many parents and children reported ‘often’ or ‘almost always’ having a problem with bathing alone (PF5; respectively, 77%, 65%), lifting heavy things (PF4; 62%, 45%), and most reported feeling tired at least ‘sometimes’ (PF8; 79%, 69%). Nearly half reported ‘often’ or ‘almost always’ having trouble sleeping (EF4; 40%, 41%), had pain (PF7; 48%, 44%). Few reported ‘often’ or ‘always’ feeling afraid/scared (EF1; 17%, 0%), sad (EF2; 7%, 0%), angry (EF3; 13%, 5%) or worried about what would happen to them (EF5; 7%, 4%). Although few reported ‘often’ or ‘always’ having a problem getting on with other children (SOF1; 24%, 8%), many had difficulty ‘often’ or ‘always’ keeping up when playing with other children (SOF5; 61%, 34%) or not being able to do things other children their age could do (SOF4; 64%, 45%). Many reported missing school at least ‘sometimes’ due to not feeling well (SCF4; 68%, 73%) or to attend medical appointments (SCF5; 82%, 80%) and they had difficulty keeping up with school activities (SCF3; 73%, 54%). They also reported at least ‘sometimes’ having difficulty paying attention in class (SCF1; 61%, 54%) although fewer reported forgetting things (SCF2; 23%, 41%).

## Discussion

This is the first study to explore in detail the impact of RDEB on HRQoL and to report findings by RDEB subtype, with more severe subtypes reporting poorer HRQoL. Our finding that individuals reported severe impact on HRQoL is similar to a few previous smaller studies in RDEB or DEB which also had QOLEB scores of around 20 [24, 36, 37]. However, other studies have demonstrated lower [34, 35, 41] or higher [3, 33, 38] average QOLEB scores, ranging between 11.7 and 35.5. The variability of scores likely represents the heterogeneity of patients within these groups, none of which were reported by RDEB subtype. Jeon et al. [3] found QOLEB severe scores for RDEB-S (*n* = 7) and RDEB-I (*n* = 6), respectively 30.14 and 23.17, although these were not statistically significantly different. In another study comparing RDEB-S (*n* = 11) to other types of RDEB (*n* = 13), both groups scored medium impact on HRQoL, respectively 15.6 and 11.1, but statistical significance was not reported [34]. Our finding of statistically significantly higher median QOLEB scores for RDEB-S participants compared to those with RDEB-I and RDEB-Inv, for total scores and functioning subscore, presumably reflects the general severity of symptoms, impact of daily living and burden of disease due to time spent on dressing changes.

Previous studies have reported opposing conclusions regarding a link between EB symptom severity and HRQoL. For example, Bruckner et al. reported a strong correlation between patient-reported severity (ISP) and QOLEB, with 16 (52%) of their participants having RDEB [47], and Tabolli et al. reported similar findings for 125 patient with EB including 39 with RDEB [23]. In contrast, Eng et al. found self-reported RDEB severity (*n* = 85), categorised as overall skin disease severity, was not correlated with HRQoL although QOLEB scores did correlate with larger wound sizes [24]. Our bigger study found large positive correlations between QOLEB (total scores and functioning subscore) and RDEB severity (iscorEB and BEBS) when considering all RDEB, demonstrating that those with more severe symptoms reported worse HRQoL; this was also significant for the subtypes RDEB-S (worse symptoms, poorer HRQoL) and RDEB-I (milder symptoms, better HRQoL). The few participants with RDEB-Pru (*n* = 4) reported worst symptom severity and highest QOLEB total scores and sub scores, including items regarding pain (Q3), frustration (Q8), anxiety (Q14) and depression (Q16), which may reflect the substantial impact of itch on QOL for this subtype due to itch frequency, severity, distress and interference with sleep [15]. Participants with RDEB-S reported the second worst QOLEB score whereas those with milder subtypes, inversa and intermediate, had milder symptoms although their QOLEB scores were still within the moderate range. We found a similar pattern for child participants and their parents/guardians who also reported worse HRQoL (PedsQL) for those with greater symptom severity; although the number of child participants was relatively small (*n* = 14), a strong association was seen when analysing PedsQL at index and all reviews.

We found that adults (QOLEB) and children (PedsQL) showed a greater impact of EB symptoms on functioning/physical health than emotions/psychosocial health, with the highest functioning/physical health impact in RDEB-S and RDEB-Pru, although the latter group also had higher emotion subscores than other subtypes. In addition, all participants with RDEB-Pru experienced anxiety (QOLEB, Q14) at index review; together, these results may reflect the psychological impact of constant, treatment-resistant extreme itch in these individuals. Similar results reported by Tabolli et al. using the Short Form-36 questionnaire in EB showed impaired physical components but no such impairment for mental components [23], whereas Martins Freitas et al. found greater impact in emotional functioning in their study of 31 adults of whom 17 had RDEB [51]. In our study, individual item responses from QOLEB highlight the physical impacts on function and finances as a result of EB, in RDEB-S in particular, with less marked impact for other subtypes. In contrast, participants generally had little or no embarrassment, discomfort due to teasing, feelings of depression, frustration or impacts on friends and family relationships. It could be postulated that relative emotional adaptation to living with RDEB might be expected as a lifelong condition that would usually present at or shortly after birth, thereby being part of what is ‘normal’ for the patient, resulting in a degree of psychological adjustment. The higher emotion scores in RDEB-Pru adults might, therefore, reflect the later development of the very itchy phenotype, and that this group is therefore psychologically more vulnerable by not having a protective effect from early onset.

Our observed larger correlation between emotions subscores (QOLEB, PedsQL) and self-reported severity scores (ISP) but not iscorEB clinician scores (ISC), except for RDEB-Pru, may highlight the contribution of subjective perceptions and expectations to HRQoL. Salamon et al. recently described a correlation between ISP and QOLEB in individuals with different types of EB including DEB [45]. Previous studies in RDEB have demonstrated correlation between body surface area affected by wounding and HRQoL, but conflicting results regarding an association between HRQoL and patient-reported disease severity measured with iscorEB or Patient Global Assessment [23, 46]. Of note, QOLEB and ISP responses report severity of impact (none, mild/a little – severe) whereas PedsQL options report frequency of event (never – almost always).

We found a negative correlation between age and QOLEB total and functioning scores, that is, worse HRQoL reported by younger adult participants, those with RDEB-S in particular, which has not been reported previously. In general, disease severity increases over time in RDEB with more chronic wounds, larger body surface area of involvement, greater SCC risk and increasing internal complications. Our results may indicate that individuals adjust to their disease over time, becoming better able to function despite increasing limitations.

Of interest, parents reported worse physical and psychosocial health (PedsQL) scores than affected children, with a greater impact on physical health noted in both groups. Parent scores for physical health showed a negative correlation with age indicating worse scores for younger children which, as clinical severity increases over time, might suggest child and parental adaptation as they live with RDEB and its treatment. Children had more difficulty with and required more assistance with physical activities, and had frequent pain, difficulty sleeping and tiredness, but being scared, sad, afraid, angry or worried affected only few children. Again, these results might indicate emotional and psychological adaptation to living with EB, whereas the physical impact of disease has a bigger negative effect on HRQoL; this has been reported elsewhere [23, 52].

### Strengths & limitations

Our study is the first to explore HRQoL in detail in different subtypes of RDEB including comparison with disease severity and by age. Comparison of data for index (baseline) reviews (*n* = 58) with all available reviews (*n* = 321) found no substantial differences in severity scores (iscorEB and BEBS) or QOL scores (QOLEB and PedsQL) during the median 6 [3, 7] years study participation, so our index findings for QOL scores can be considered representative of the RDEB population and subtypes. Limitations of our study include a relative underrepresentation of children in our cohort and smaller numbers of rarer RDEB subtypes. Although QOLEB has been validated in adults with different types of EB, this tool has not been specifically validated across different subtypes of RDEB. We note the recent recommendation to use QOLEB for children aged 10 years and older, with dermatology-specific tools used in addition to ISP for children under 10 years [53].

## Conclusion

We report HRQoL in a large cohort of individuals with RDEB by age and disease subtype. Using QOLEB as a disease-specific tool in adult participants, HRQoL was severely impacted for all RDEB, and particularly high in RDEB-Pru (albeit with small patient numbers), and RDEB-S who had statistically significant worse HRQoL compared to RDEB-I and RDEB-Inv. Total and functioning QOLEB scores correlated with disease severity for all RDEB as measured with iscorEB and BEBS. In children, similar results were observed, with worse HRQoL captured with PedsQL correlating with higher iscorEB scores. Interestingly, functioning/physical health in both adults and children was more severely impacted than emotions/psychosocial health suggesting a degree of psychological adaptation from having RDEB, a lifelong condition presenting at or shortly after birth, therefore representing what is ‘normal’ for that individual. Relative improvement in HRQoL with age, despite a general worsening of disease severity and complications over time, might suggest, however, that individuals with RDEB continue to adapt throughout life with a diminution of impact over time.

## Supplementary Information

Below is the link to the electronic supplementary material.

Supplementary Material 1

Supplementary Material 2

Supplementary Material 3

Supplementary Material 4

Supplementary Material 5

Supplementary Material 6

## Data Availability

The datasets generated and analysed during the current study are not publicly available as the authors intend to prepare further publications from them. However, the authors would consider reasonable requests to access the data and will make these available in an accessible repository once all relevant data has been published.

## Abbreviations

BEBS: Birmingham Epidermolysis Bullosa Severity score
EB: Epidermolysis bullosa
DEB: Dystrophic epidermolysis bullosa
HRQoL: Health-related quality of life
IQR: Interquartile range [1st quartile, 3rd quartile]
ISC: iscorEB clinician score
ISP: iscorEB patient score
iscorEB: Instrument for Scoring Clinical Outcomes for Epidermolysis Bullosa
QoL: Quality of life
QOLEB: Quality of Life in Epidermolysis Bullosa questionnaire
PEBLES: The Prospective Epidermolysis Bullosa Longitudinal Evaluation Study
PedsQL: Pediatric Quality of Life Inventory, version 4.0 generic core scales
RDEB: Recessive dystrophic epidermolysis bullosa
RDEB-I: Intermediate RDEB
RDEB-Inv: RDEB inversa
RDEB-Pru: RDEB pruriginosa
RDEB-PT: Pretibial RDEB
RDEB-S: Severe RDEB
SCC: Squamous cell carcinoma
