# Supplementary material for: Health-related quality of life in recessive dystrophic epidermolysis bullosa: findings of the Prospective Epidermolysis Bullosa Longitudinal Evaluation Study (PEBLES)
Source: Orphanet J Rare Dis. 2026 May 6;21:177. doi: 10.1186/s13023-026-04330-5 (PMC13147842; doi:10.1186/s13023-026-04330-5)
Supplement: Supplementary file 1 — Supplementary Material 1 [file 13023_2026_4330_MOESM1_ESM.docx]

### Additional file 1: Severity scores for all reviews (n=335)

| Variable | All RDEB^1^ | RDEB-S | RDEB-I | RDEB-Inv | RDEB-Pru |
| --- | --- | --- | --- | --- | --- |
| n | 335 | 166 | 99 | 51 | 14 |
| iscorEB total score^2^ | 66 [46,89] (n = 223) | 74 [60,96] (n = 110) | 48 [33,70] (n = 63) | 46 [37,61] (n = 37) | 90 [80,97] (n = 12) |
| ISC clinician score^2^ | 21 [10,32] (n = 236) | 30 [21,39] (n = 122) | 10 [7,21] (n = 64) | 7 [5,11] (n = 37) | 23 [18,28] (n = 12) |
| ISP patient score^2^ | 44 [28,56] (n = 316) | 46 [36,56] (n = 151) | 28 [12,54] (n = 97) | 36 [24,52] (n = 50) | 65 [60,73] (n = 13) |
| BEBS total score^3^ | 25 [13,37] (n = 308) | 36 [28,45] (n = 148) | 14 [6,23] (n = 93) | 12 [8,16] (n = 49) | 27 [22,34] (n = 13) |
| Annual dressing time, hrs | 364 [91,637] (n = 293) | 546 [364,910] (n = 165) | 61 [18,182] (n = 81) | 61 [12,121] (n = 28) | 572 [370,1547] (n = 14) |

*Results are presented as median [IQR] (number) or number (%).*

*S=RDEB severe (RDEB-S), I=intermediate (RDEB-I), Inv=inversa (RDEB-Inv), Pru=pruriginosa (RDEB-Pru)^1^ Reviews by one individual with pretibial RDEB (n=5) were included in overall analysis but not separately reported.*

*^2^ Instrument for scoring clinical outcomes of research for epidermolysis bullosa (iscorEB), maximum clinician (ISC) score of 138 and self-reported (ISP) score of 120; higher scores = worse severity.*

*^3^ Birmingham Epidermolysis Bullosa Severity (BEBS) score, maximum of 100; higher scores = worse severity.*
