# Supplementary material for: Health-related quality of life in recessive dystrophic epidermolysis bullosa: findings of the Prospective Epidermolysis Bullosa Longitudinal Evaluation Study (PEBLES)
Source: Orphanet J Rare Dis. 2026 May 6;21:177. doi: 10.1186/s13023-026-04330-5 (PMC13147842; doi:10.1186/s13023-026-04330-5)
Supplement: Supplementary file 2 — Supplementary Material 2 [file 13023_2026_4330_MOESM2_ESM.docx]

### Additional file 2: Pairwise comparisons of QOLEB^1^ scores between RDEB subtypes at index review

|  | | | QOLEB score | | |
| --- | --- | --- | --- | --- | --- |
| P-values | Group 1 | Group 2 | Functioning | Emotions | Total |
| Unadjusted | RDEB-S | RDEB-I | 0.001 | 0.273 | 0.001 |
|  | RDEB-S | RDEB-Inv | 0.005 | 0.631 | 0.007 |
|  | RDEB-S | RDEB-Pru | 0.881 | 0.106 | 0.802 |
|  | RDEB-I | RDEB-Inv | 0.625 | 0.533 | 0.680 |
|  | RDEB-I | RDEB-Pru | 0.105 | 0.023 | 0.055 |
|  | RDEB-Inv | RDEB-Pru | 0.216 | 0.029 | 0.121 |
| Adjusted | RDEB-S | RDEB-I | 0.004 | 0.410 | 0.008 |
|  | RDEB-S | RDEB-Inv | 0.015 | 0.631 | 0.020 |
|  | RDEB-S | RDEB-Pru | 0.881 | 0.212 | 0.802 |
|  | RDEB-I | RDEB-Inv | 0.750 | 0.631 | 0.802 |
|  | RDEB-I | RDEB-Pru | 0.210 | 0.086 | 0.110 |
|  | RDEB-Inv | RDEB-Pru | 0.324 | 0.086 | 0.182 |

*Results are presented as unadjusted and adjusted p-values.*

*S=RDEB severe (RDEB-S), I=intermediate (RDEB-I), Inv=inversa (RDEB-Inv), Pru=pruriginosa (RDEB-Pru)*

*Outcomes were compared using the Mann-Whitney U test. P-values were adjusted using the Benjamini-Hochberg procedure. P-values that are significant (p<0.05) or approaching significance (p~0.05) are indicated in green and blue, respectively.*

*^1^ Quality of Life in Epidermolysis Bullosa (QOLEB)*
