# Supplementary material for: Health-related quality of life in recessive dystrophic epidermolysis bullosa: findings of the Prospective Epidermolysis Bullosa Longitudinal Evaluation Study (PEBLES)
Source: Orphanet J Rare Dis. 2026 May 6;21:177. doi: 10.1186/s13023-026-04330-5 (PMC13147842; doi:10.1186/s13023-026-04330-5)
Supplement: Supplementary file 3 — Supplementary Material 3 [file 13023_2026_4330_MOESM3_ESM.docx]

### Additional file 3: QOL scores (QOLEB^1^, PedsQL^2^) at all reviews (n=335)

| Variable | All RDEB^3^ | RDEB-S | RDEB-I | RDEB-Inv | RDEB-Pru |
| --- | --- | --- | --- | --- | --- |
| **QOLEB**, n | 244 | 81 | 93 | 51 | 14 |
| QOLEB score | 18 [13,26] (n = 240) | 24 [19,31] (n = 81) | 14 [6,21] (n = 91) | 15 [11,20] (n = 49) | 30 [26,35] (n = 14) |
| QOLEB Functioning subscore | 14 [8,20] (n = 241) | 19 [16,23] (n = 81) | 9 [5,14] (n = 92) | 10 [6,14] (n = 49) | 22 [18,25] (n = 14) |
| QOLEB emotions subscore | 5 [3,7] (n = 244) | 5 [3,8] (n = 81) | 5 [1,6] (n = 93) | 4 [4,6] (n = 51) | 8 [8,9] (n = 14) |
| **PedsQL**, n | 89 | 84 | 5 | 0 | 0 |
| PedsQL total score, parent | 48 [39,56] (n = 88) | 48 [39,55] (n = 83) | 57 [57,67] (n = 5) |  |  |
| PedsQL total score, patient | 54 [46,61] (n = 63) | 54 [46,61] (n = 61) | 57 [56,58] (n = 2) |  |  |
| PedsQL physical health, parent | 34 [19,47] (n = 89) | 33 [19,47] (n = 84) | 47 [44,66] (n = 5) |  |  |
| PedsQL physical health, patient | 41 [25,53] (n = 64) | 38 [25,53] (n = 62) | 48 [48,49] (n = 2) |  |  |
| PedsQL psychsocial health, parent | 55 [48,65] (n = 88) | 55 [47,65] (n = 83) | 63 [63,68] (n = 5) |  |  |
| PedsQL psychsocial health, patient | 62 [54,70] (n = 63) | 62 [53,70] (n = 61) | 62 [59,64] (n = 2) |  |  |

*Results are presented as median [IQR] (number).*

*S=RDEB severe (RDEB-S), I=intermediate (RDEB-I), Inv=inversa (RDEB-Inv), Pru=pruriginosa (RDEB-Pru)*

*^1-^ Quality of Life in Epidermolysis Bullosa (QOLEB) maximum score of 51, includes functioning subscore of 36 and emotions subscore of 15; higher scores = worse HRQoL*

*^2^ Pediatric Quality of Life Inventory (PedsQL), total score maximum of 100; includes physical health summary score (“physical health”) maximum of 100; psychosocial health summary score (“psychosocial health”) maximum of 100; higher scores=better HRQoL*

*^3^Reviews by one individual with pretibial RDEB (n=5) were included in overall analysis but not separately reported.*
