# Supplementary material for: Health-related quality of life in recessive dystrophic epidermolysis bullosa: findings of the Prospective Epidermolysis Bullosa Longitudinal Evaluation Study (PEBLES)
Source: Orphanet J Rare Dis. 2026 May 6;21:177. doi: 10.1186/s13023-026-04330-5 (PMC13147842; doi:10.1186/s13023-026-04330-5)
Supplement: Supplementary file 4 — Supplementary Material 4 [file 13023_2026_4330_MOESM4_ESM.docx]

### Additional file 4: responses to QOLEB^1^ items by RDEB subtype at index review (n=47)

| Variable | | Category | All RDEB^2^ | RDEB-S | | RDEB-I | | RDEB-Inv | | RDEB-Pru | |  |
| --- | --- | --- | --- | --- | --- | --- | --- | --- | --- | --- | --- | --- |
| Q1: Movement at home | | Not at all | 17 (36) | 3 (20) | | 10 (56) | | 3 (33) | | 1 (25) | |  |
|  |  | A little | 16 (34) | 6 (40) | | 5 (28) | | 4 (44) | | 1 (25) | |  |
|  |  | A lot | 8 (17) | 1 (7) | | 3 (17) | | 2 (22) | | 1 (25) | |  |
|  |  | Severely | 6 (13) | 5 (33) | | 0 (0) | | 0 (0) | | 1 (25) | |  |
| Q2: Bathing | | No impact | 19 (40) | 1 (7) | | 12 (67) | | 6 (67) | | 0 (0) | |  |
|  | | Some assistance | 11 (23) | 3 (20) | | 4 (22) | | 1 (11) | | 2 (50) | |  |
|  | | Assist most times | 2 (4) | 0 (0) | | 0 (0) | | 2 (22) | | 0 (0) | |  |
|  | | Assist all times | 15 (32) | 11 (73) | | 2 (11) | | 0 (0) | | 2 (50) | |  |
| Q3: Pain | | No pain | 5 (11) | 0 (0) | | 3 (17) | | 1 (11) | | 1 (25) | |  |
|  | | Occasional pain | 16 (34) | 5 (33) | | 7 (39) | | 4 (44) | | 0 (0) | |  |
|  | | Frequent pain | 13 (28) | 6 (40) | | 4 (22) | | 2 (22) | | 1 (25) | |  |
|  | | Constant pain | 13 (28) | 4 (27) | | 4 (22) | | 2 (22) | | 2 (50) | |  |
| Q4: Writing | | No interference | 27 (57) | 3 (20) | | 14 (78) | | 6 (67) | | 3 (75) | |  |
|  | | Difficult to grip | 6 (13) | 3 (20) | | 2 (11) | | 1 (11) | | 0 (0) | |  |
|  | | Easier to type | 13 (28) | 8 (53) | | 2 (11) | | 2 (22) | | 1 (25) | |  |
|  | | Cannot write | 1 (2) | 1 (7) | | 0 (0) | | 0 (0) | | 0 (0) | |  |
| Q5: Eating | | No, eat normally | 10 (21) | 0 (0) | | 7 (39) | | 1 (11) | | 1 (25) | |  |
|  | | A little | 17 (36) | 8 (53) | | 5 (28) | | 2 (22) | | 2 (50) | |  |
|  | | A lot | 18 (38) | 5 (33) | | 6 (33) | | 6 (67) | | 1 (25) | |  |
|  | | Use gastro tube | 2 (4) | 2 (13) | | 0 (0) | | 0 (0) | | 0 (0) | |  |
| Q6: Shopping | | Not at all | 15 (32) | 0 (0) | | 8 (44) | | 6 (67) | | 1 (25) | |  |
|  | | A little | 12 (26) | 3 (20) | | 6 (33) | | 2 (22) | | 0 (0) | |  |
|  | | A lot | 9 (19) | 2 (13) | | 4 (22) | | 1 (11) | | 2 (50) | |  |
|  | | Need assist all | 11 (23) | 10 (67) | | 0 (0) | | 0 (0) | | 1 (25) | |  |
| Q7: Sports | | No impact | 4 (9) | 0 (0) | | 2 (11) | | 1 (11) | | 0 (0) | |  |
|  | | Cautious | 4 (9) | 1 (7) | | 3 (17) | | 0 (0) | | 0 (0) | |  |
|  | | Avoid some | 19 (40) | 3 (20) | | 9 (50) | | 6 (67) | | 1 (25) | |  |
|  | | Avoid all | 20 (43) | 11 (73) | | 4 (22) | | 2 (22) | | 3 (75) | |  |
| Q8: Frustration | | No frustration | 4 (9) | 0 (0) | | 4 (22) | | 0 (0) | | 0 (0) | |  |
|  | | A little | 21 (45) | 8 (53) | | 9 (50) | | 4 (44) | | 0 (0) | |  |
|  | | A lot | 17 (36) | 5 (33) | | 5 (28) | | 4 (44) | | 3 (75) | |  |
|  | | Angry all the time | 5 (11) | 2 (13) | | 0 (0) | | 1 (11) | | 1 (25) | |  |
| Q9: Movement outside | | No a little | 9 (19) | 1 (7) | | 4 (22) | | 3 (33) | | 1 (25) | |  |
|  |  | A little | 18 (38) | 5 (33) | | 9 (50) | | 4 (44) | | 0 (0) | |  |
|  |  | A lot | 16 (34) | 6 (40) | | 5 (28) | | 2 (22) | | 2 (50) | |  |
|  |  | Severely | 4 (9) | 3 (20) | | 0 (0) | | 0 (0) | | 1 (25) | |  |
| Q10: Family relationships | | No impact | 17 (36) | 4 (27) | | 9 (50) | | 3 (33) | | 1 (25) | |  |
|  |  | Small impact | 13 (28) | 4 (27) | | 5 (28) | | 4 (44) | | 0 (0) | |  |
|  |  | Large impact | 8 (17) | 2 (13) | | 2 (11) | | 1 (11) | | 2 (50) | |  |
|  |  | Very large impact | 9 (19) | 5 (33) | | 2 (11) | | 1 (11) | | 1 (25) | |  |
| Q11: Embarrassment | | None | 12 (26) | 5 (33) | | 3 (17) | | 4 (44) | | 0 (0) | |  |
|  |  | A little | 26 (55) | 6 (40) | | 11 (61) | | 5 (56) | | 3 (75) | |  |
|  |  | A lot | 8 (17) | 4 (27) | | 4 (22) | | 0 (0) | | 0 (0) | |  |
|  | | Extremely | 1 (2) | 0 (0) | | 0 (0) | | 0 (0) | | 1 (25) | |  |
| Q12: Home modifications | | No, not at all | 25 (53) | 5 (33) | | 12 (67) | | 7 (78) | | 1 (25) | |  |
|  |  | A few | 15 (32) | 8 (53) | | 4 (22) | | 0 (0) | | 2 (50) | |  |
|  |  | A lot | 6 (13) | 1 (7) | | 2 (11) | | 2 (22) | | 1 (25) | |  |
|  |  | Extensive | 1 (2) | 1 (7) | | 0 (0) | | 0 (0) | | 0 (0) | |  |
| Q13: Friendships | Not at all | | 20 (43) | | 6 (40) | | 10 (56) | | 4 (44) | | 0 (0) | |
|  | A little | | 16 (34) | | 3 (20) | | 6 (33) | | 5 (56) | | 1 (25) | |
|  | A lot | | 6 (13) | | 2 (13) | | 2 (11) | | 0 (0) | | 2 (50) | |
|  | Restricts socialising | | 5 (11) | | 4 (27) | | 0 (0) | | 0 (0) | | 1 (25) | |
| Q14: Anxiety | Not anxious | | 8 (17) | | 1 (7) | | 6 (33) | | 1 (11) | | 0 (0) | |
|  | A little | | 18 (38) | | 7 (47) | | 7 (39) | | 3 (33) | | 0 (0) | |
|  | A lot | | 19 (40) | | 6 (40) | | 5 (28) | | 5 (56) | | 3 (75) | |
|  | Extremely | | 2 (4) | | 1 (7) | | 0 (0) | | 0 (0) | | 1 (25) | |
| Q15: Financial impact | No impact | | 11 (23) | | 0 (0) | | 8 (44) | | 1 (11) | | 1 (25) | |
|  | Slightly affected | | 13 (28) | | 3 (20) | | 3 (17) | | 5 (56) | | 2 (50) | |
|  | Greatly affected | | 10 (21) | | 5 (33) | | 3 (17) | | 2 (22) | | 0 (0) | |
|  | Severely affected | | 13 (28) | | 7 (47) | | 4 (22) | | 1 (11) | | 1 (25) | |
| Q16: Depression | Not depressed | | 11 (23) | | 3 (20) | | 6 (33) | | 2 (22) | | 0 (0) | |
|  | A little | | 19 (40) | | 6 (40) | | 9 (50) | | 4 (44) | | 0 (0) | |
|  | A lot | | 15 (32) | | 5 (33) | | 3 (17) | | 2 (22) | | 4 (100) | |
|  | Constant depression | | 2 (4) | | 1 (7) | | 0 (0) | | 1 (11) | | 0 (0) | |
| Q17: Uncomfortable | Not at all | | 15 (32) | | 3 (20) | | 6 (33) | | 5 (56) | | 0 (0) | |
|  | A little | | 25 (53) | | 10 (67) | | 9 (50) | | 4 (44) | | 2 (50) | |
|  | A lot | | 7 (15) | | 2 (13) | | 3 (17) | | 0 (0) | | 2 (50) | |
|  | Don't go out | | 0 (0) | | 0 (0) | | 0 (0) | | 0 (0) | | 0 (0) | |

*Results are presented as number (%).*

*S=RDEB severe (RDEB-S), I=intermediate (RDEB-I), Inv=inversa (RDEB-Inv), Pru=pruriginosa (RDEB-Pru)^1^ ^1^Quality of Life in Epidermolysis Bullosa (QOLEB)*

*^2^Reviews by one individual with pretibial RDEB (n=5) were included in overall analysis but not separately reported.*
