# Supplementary material for: Health-related quality of life in recessive dystrophic epidermolysis bullosa: findings of the Prospective Epidermolysis Bullosa Longitudinal Evaluation Study (PEBLES)
Source: Orphanet J Rare Dis. 2026 May 6;21:177. doi: 10.1186/s13023-026-04330-5 (PMC13147842; doi:10.1186/s13023-026-04330-5)
Supplement: Supplementary file 5 — Supplementary Material 5 [file 13023_2026_4330_MOESM5_ESM.docx]

### Additional file 5: responses to QOLEB^1^ items by RDEB subtype for all reviews (n=245)

| Variable | Category | All RDEB^2^ | RDEB-S | RDEB-I | RDEB-Inv | RDEB-Pru |
| --- | --- | --- | --- | --- | --- | --- |
| Q1: Movement at home | Not at all | 87 (36) | 16 (20) | 51 (54) | 19 (37) | 1 (7) |
|  | A little | 106 (43) | 41 (51) | 37 (39) | 22 (43) | 3 (21) |
|  | A lot | 37 (15) | 14 (17) | 6 (6) | 9 (18) | 6 (43) |
|  | Severely | 15 (6) | 10 (12) | 0 (0) | 1 (2) | 4 (29) |
| Q2: Bathing | No impact | 102 (42) | 5 (6) | 60 (64) | 34 (68) | 0 (0) |
|  | Some assistance | 53 (22) | 13 (16) | 25 (27) | 8 (16) | 5 (36) |
|  | Assist most times | 19 (8) | 6 (7) | 3 (3) | 6 (12) | 4 (29) |
|  | Assist all times | 70 (29) | 57 (70) | 6 (6) | 2 (4) | 5 (36) |
| Q3: Pain | No pain | 19 (8) | 0 (0) | 15 (16) | 3 (6) | 1 (7) |
|  | Occasional pain | 90 (37) | 29 (36) | 40 (43) | 20 (39) | 0 (0) |
|  | Frequent pain | 71 (29) | 29 (36) | 24 (26) | 14 (27) | 2 (14) |
|  | Constant pain | 65 (27) | 23 (28) | 15 (16) | 14 (27) | 11 (79) |
| Q4: Writing | No interference | 135 (55) | 19 (23) | 63 (68) | 37 (73) | 11 (79) |
|  | Difficult to grip | 42 (17) | 17 (21) | 16 (17) | 7 (14) | 2 (14) |
|  | Easier to type | 61 (25) | 39 (48) | 14 (15) | 7 (14) | 1 (7) |
|  | Cannot write | 6 (2) | 6 (7) | 0 (0) | 0 (0) | 0 (0) |
| Q5: Eating | No, eat normally | 54 (22) | 6 (7) | 33 (35) | 6 (12) | 4 (29) |
|  | A little | 97 (40) | 44 (54) | 24 (26) | 21 (41) | 8 (57) |
|  | A lot | 91 (37) | 28 (35) | 37 (39) | 24 (47) | 2 (14) |
|  | Use gastro tube | 3 (1) | 3 (4) | 0 (0) | 0 (0) | 0 (0) |
| Q6: Shopping | Not at all | 77 (31) | 7 (9) | 44 (47) | 24 (47) | 1 (7) |
|  | A little | 73 (30) | 16 (20) | 35 (37) | 16 (31) | 2 (14) |
|  | A lot | 45 (18) | 19 (23) | 11 (12) | 7 (14) | 8 (57) |
|  | Need assist all | 50 (20) | 39 (48) | 4 (4) | 4 (8) | 3 (21) |
| Q7: Sports | No impact | 23 (9) | 1 (1) | 13 (14) | 6 (12) | 0 (0) |
|  | Cautious | 40 (16) | 2 (2) | 23 (24) | 14 (29) | 0 (0) |
|  | Avoid some | 72 (30) | 17 (21) | 34 (36) | 18 (37) | 2 (14) |
|  | Avoid all | 108 (44) | 61 (75) | 24 (26) | 11 (22) | 12 (86) |
| Q8: Frustration | No frustration | 34 (14) | 4 (5) | 30 (32) | 0 (0) | 0 (0) |
|  | A little | 117 (48) | 40 (49) | 42 (45) | 31 (61) | 2 (14) |
|  | A lot | 79 (32) | 31 (38) | 18 (19) | 17 (33) | 11 (79) |
|  | Angry all the time | 15 (6) | 6 (7) | 4 (4) | 3 (6) | 1 (7) |
| Q9: Movement outside | No a little | 51 (21) | 5 (6) | 29 (31) | 16 (31) | 1 (7) |
|  | A little | 103 (42) | 26 (32) | 47 (50) | 26 (51) | 1 (7) |
|  | A lot | 64 (26) | 32 (40) | 17 (18) | 7 (14) | 6 (43) |
|  | Severely | 27 (11) | 18 (22) | 1 (1) | 2 (4) | 6 (43) |
| Q10: Family relationships | No impact | 89 (36) | 21 (26) | 47 (50) | 15 (29) | 2 (14) |
|  | Small impact | 97 (40) | 32 (40) | 29 (31) | 32 (63) | 4 (29) |
|  | Large impact | 36 (15) | 16 (20) | 11 (12) | 2 (4) | 6 (43) |
|  | Very large impact | 23 (9) | 12 (15) | 7 (7) | 2 (4) | 2 (14) |
| Q11: Embarrassment | None | 100 (41) | 35 (43) | 34 (36) | 26 (51) | 1 (7) |
|  | A little | 112 (46) | 37 (46) | 43 (46) | 23 (45) | 8 (57) |
|  | A lot | 29 (12) | 8 (10) | 17 (18) | 1 (2) | 3 (21) |
|  | Extremely | 4 (2) | 1 (1) | 0 (0) | 1 (2) | 2 (14) |
| Q12: Home modifications | No, not at all | 142 (58) | 30 (37) | 71 (76) | 37 (73) | 1 (7) |
|  | A few | 64 (26) | 33 (41) | 17 (18) | 4 (8) | 8 (57) |
|  | A lot | 23 (9) | 6 (7) | 5 (5) | 9 (18) | 3 (21) |
|  | Extensive | 16 (7) | 12 (15) | 1 (1) | 1 (2) | 2 (14) |
| Q13: Friendships | Not at all | 126 (51) | 39 (48) | 57 (61) | 25 (49) | 1 (7) |
|  | A little | 83 (34) | 22 (27) | 32 (34) | 22 (43) | 6 (43) |
|  | A lot | 24 (10) | 13 (16) | 5 (5) | 2 (4) | 4 (29) |
|  | Restricts socialising | 12 (5) | 7 (9) | 0 (0) | 2 (4) | 3 (21) |
| Q14: Anxiety | Not anxious | 50 (20) | 14 (17) | 33 (35) | 1 (2) | 0 (0) |
|  | A little | 100 (41) | 39 (48) | 26 (28) | 30 (59) | 2 (14) |
|  | A lot | 78 (32) | 22 (27) | 29 (31) | 19 (37) | 8 (57) |
|  | Extremely | 16 (7) | 6 (7) | 5 (5) | 1 (2) | 4 (29) |
| Q15: Financial impact | No impact | 65 (27) | 4 (5) | 43 (46) | 12 (24) | 1 (7) |
|  | Slightly affected | 75 (31) | 23 (28) | 19 (20) | 27 (53) | 6 (43) |
|  | Greatly affected | 53 (22) | 25 (31) | 16 (17) | 6 (12) | 6 (43) |
|  | Severely affected | 51 (21) | 29 (36) | 15 (16) | 6 (12) | 1 (7) |
| Q16: Depression | Not depressed | 75 (31) | 21 (26) | 44 (47) | 8 (16) | 0 (0) |
|  | A little | 104 (42) | 36 (44) | 35 (37) | 30 (59) | 2 (14) |
|  | A lot | 53 (22) | 16 (20) | 12 (13) | 11 (22) | 12 (86) |
|  | Constant depression | 13 (5) | 8 (10) | 3 (3) | 2 (4) | 0 (0) |
| Q17: Uncomfortable | Not at all | 112 (46) | 31 (38) | 40 (43) | 35 (69) | 1 (7) |
|  | A little | 114 (47) | 43 (53) | 46 (49) | 15 (29) | 10 (71) |
|  | A lot | 17 (7) | 6 (7) | 8 (9) | 0 (0) | 3 (21) |
|  | Don't go out | 2 (1) | 1 (1) | 0 (0) | 1 (2) | 0 (0) |

*Results are presented as number (%).*

*S=RDEB severe (RDEB-S), I=intermediate (RDEB-I), Inv=inversa (RDEB-Inv), Pru=pruriginosa (RDEB-Pru)*

*^1^Quality of Life in Epidermolysis Bullosa (QOLEB)*

*^2^Reviews by one individual with pretibial RDEB (n=5) were included in overall analysis but not separately reported.*
