# Supplementary material for: Health-related quality of life in recessive dystrophic epidermolysis bullosa: findings of the Prospective Epidermolysis Bullosa Longitudinal Evaluation Study (PEBLES)
Source: Orphanet J Rare Dis. 2026 May 6;21:177. doi: 10.1186/s13023-026-04330-5 (PMC13147842; doi:10.1186/s13023-026-04330-5)
Supplement: Supplementary file 6 — Supplementary Material 6 [file 13023_2026_4330_MOESM6_ESM.docx]

### Additional file 6: PedsQL^1^ responses by RDEB subtype, all reviews (n=89)

| Variable | Category | All RDEB Parent | RDEB-S Parent | | RDEB-I Parent | | All RDEB Child | | RDEB-S Child | | RDEB-I Child | |  |
| --- | --- | --- | --- | --- | --- | --- | --- | --- | --- | --- | --- | --- | --- |
| PF1: Walking | Never | 2 (2) | 1 (1) | | 1 (20) | | 1 (2) | | 1 (2) | | 0 (0) | |  |
|  | Almost never | 17 (19) | 14 (17) | | 3 (60) | | 10 (16) | | 8 (13) | | 2 (100) | |  |
|  | Sometimes | 39 (44) | 39 (46) | | 0 (0) | | 32 (50) | | 32 (52) | | 0 (0) | |  |
|  | Often | 13 (15) | 12 (14) | | 1 (20) | | 4 (6) | | 4 (6) | | 0 (0) | |  |
|  | Almost always | 18 (20) | 18 (21) | | 0 (0) | | 17 (27) | | 17 (27) | | 0 (0) | |  |
| PF2: Running | Never | 1 (1) | 0 (0) | | 1 (20) | | 2 (3) | | 2 (3) | | 0 (0) | |  |
|  | Almost never | 4 (4) | 3 (4) | | 1 (20) | | 5 (8) | | 4 (6) | | 1 (50) | |  |
|  | Sometimes | 30 (34) | 28 (33) | | 2 (40) | | 33 (52) | | 32 (52) | | 1 (50) | |  |
|  | Often | 25 (28) | 24 (29) | | 1 (20) | | 5 (8) | | 5 (8) | | 0 (0) | |  |
|  | Almost always | 29 (33) | 29 (35) | | 0 (0) | | 19 (30) | | 19 (31) | | 0 (0) | |  |
| PF3: Sports activity | Never | 2 (2) | 1 (1) | | 1 (20) | | 5 (8) | | 5 (8) | | 0 (0) | |  |
|  | Almost never | 3 (3) | 2 (2) | | 1 (20) | | 4 (6) | | 4 (6) | | 0 (0) | |  |
|  | Sometimes | 26 (29) | 25 (30) | | 1 (20) | | 26 (41) | | 24 (39) | | 2 (100) | |  |
|  | Often | 28 (31) | 26 (31) | | 2 (40) | | 9 (14) | | 9 (15) | | 0 (0) | |  |
|  | Almost always | 30 (34) | 30 (36) | | 0 (0) | | 20 (31) | | 20 (32) | | 0 (0) | |  |
| PF4: Lifting | Never | 3 (3) | 2 (2) | | 1 (20) | | 4 (6) | | 4 (6) | | 0 (0) | |  |
|  | Almost never | 10 (11) | 9 (11) | | 1 (20) | | 9 (14) | | 9 (15) | | 0 (0) | |  |
|  | Sometimes | 20 (22) | 20 (24) | | 0 (0) | | 22 (34) | | 21 (34) | | 1 (50) | |  |
|  | Often | 12 (13) | 10 (12) | | 2 (40) | | 8 (12) | | 7 (11) | | 1 (50) | |  |
|  | Almost always | 44 (49) | 43 (51) | | 1 (20) | | 21 (33) | | 21 (34) | | 0 (0) | |  |
| PF5: Bathing | Never | 9 (11) | 7 (9) | | 2 (40) | | 3 (5) | | 3 (5) | | 0 (0) | |  |
|  | Almost never | 7 (8) | 6 (8) | | 1 (20) | | 2 (3) | | 1 (2) | | 1 (50) | |  |
|  | Sometimes | 4 (5) | 4 (5) | | 0 (0) | | 17 (27) | | 17 (27) | | 0 (0) | |  |
|  | Often | 9 (11) | 8 (10) | | 1 (20) | | 6 (9) | | 5 (8) | | 1 (50) | |  |
|  | Almost always | 56 (66) | 55 (69) | | 1 (20) | | 36 (56) | | 36 (58) | | 0 (0) | |  |
| PF6: Doing chores | Never | 10 (11) | 8 (10) | | 2 (40) | | 13 (20) | | 13 (21) | | 0 (0) | |  |
|  | Almost never | 14 (16) | 13 (15) | | 1 (20) | | 3 (5) | | 2 (3) | | 1 (50) | |  |
|  | Sometimes | 33 (37) | 32 (38) | | 1 (20) | | 30 (47) | | 29 (47) | | 1 (50) | |  |
|  | Often | 19 (21) | 18 (21) | | 1 (20) | | 8 (12) | | 8 (13) | | 0 (0) | |  |
|  | Almost always | 13 (15) | 13 (15) | | 0 (0) | | 10 (16) | | 10 (16) | | 0 (0) | |  |
| PF7: Aches/Pains | Never | 2 (2) | 1 (1) | | 1 (20) | | 2 (3) | | 2 (3) | | 0 (0) | |  |
|  | Almost never | 10 (11) | 10 (12) | | 0 (0) | | 5 (8) | | 5 (8) | | 0 (0) | |  |
|  | Sometimes | 34 (38) | 32 (38) | | 2 (40) | | 29 (45) | | 29 (47) | | 0 (0) | |  |
|  | Often | 25 (28) | 23 (27) | | 2 (40) | | 12 (19) | | 10 (16) | | 2 (100) | |  |
|  | Almost always | 18 (20) | 18 (21) | | 0 (0) | | 16 (25) | | 16 (26) | | 0 (0) | |  |
| PF8: Feeling tired | Never | 3 (3) | 3 (4) | | 0 (0) | | 17 (27) | | 17 (27) | | 0 (0) | |  |
|  | Almost never | 16 (18) | 16 (19) | | 0 (0) | | 3 (5) | | 3 (5) | | 0 (0) | |  |
|  | Sometimes | 40 (45) | 37 (44) | | 3 (60) | | 32 (50) | | 31 (50) | | 1 (50) | |  |
|  | Often | 22 (25) | 21 (25) | | 1 (20) | | 7 (11) | | 7 (11) | | 0 (0) | |  |
|  | Almost always | 8 (9) | 7 (8) | | 1 (20) | | 5 (8) | | 4 (6) | | 1 (50) | |  |
| EF1: Feeling afraid | Never | 6 (7) | 5 (6) | | 1 (20) | | 12 (19) | | 11 (18) | | 1 (50) | |  |
|  | Almost Never | 17 (19) | 17 (20) | | 0 (0) | | 12 (19) | | 12 (20) | | 0 (0) | |  |
|  | Sometimes | 50 (57) | 48 (58) | | 2 (40) | | 39 (62) | | 38 (62) | | 1 (50) | |  |
|  | Often | 14 (16) | 12 (14) | | 2 (40) | | 0 (0) | | 0 (0) | | 0 (0) | |  |
|  | Almost Always | 1 (1) | 1 (1) | | 0 (0) | | 0 (0) | | 0 (0) | | 0 (0) | |  |
| EF2: Feeling sad | Never | 9 (10) | 8 (10) | | 1 (20) | | 15 (24) | | 14 (23) | | 1 (50) | |  |
|  | Almost Never | 28 (32) | 26 (32) | | 2 (40) | | 12 (19) | | 12 (20) | | 0 (0) | |  |
|  | Sometimes | 44 (51) | 43 (52) | | 1 (20) | | 36 (57) | | 35 (57) | | 1 (50) | |  |
|  | Often | 6 (7) | 5 (6) | | 1 (20) | | 0 (0) | | 0 (0) | | 0 (0) | |  |
|  | Almost Always | 0 (0) | 0 (0) | | 0 (0) | | 0 (0) | | 0 (0) | | 0 (0) | |  |
| EF3: Feeling angry | Never | 9 (10) | 9 (11) | | 0 (0) | | 11 (17) | | 11 (18) | | 0 (0) | |  |
|  | Almost Never | 24 (27) | 23 (28) | | 1 (20) | | 7 (11) | | 6 (10) | | 1 (50) | |  |
|  | Sometimes | 43 (49) | 41 (49) | | 2 (40) | | 42 (67) | | 41 (67) | | 1 (50) | |  |
|  | Often | 11 (12) | 9 (11) | | 2 (40) | | 2 (3) | | 2 (3) | | 0 (0) | |  |
|  | Almost Always | 1 (1) | 1 (1) | | 0 (0) | | 1 (2) | | 1 (2) | | 0 (0) | |  |
| EF4: Trouble sleeping | Never | 10 (11) | 9 (11) | | 1 (20) | | 8 (13) | | 8 (13) | | 0 (0) | |  |
|  | Almost Never | 13 (15) | 11 (13) | | 2 (40) | | 3 (5) | | 3 (5) | | 0 (0) | |  |
|  | Sometimes | 30 (34) | 29 (35) | | 1 (20) | | 26 (41) | | 25 (41) | | 1 (50) | |  |
|  | Often | 13 (15) | 12 (14) | | 1 (20) | | 10 (16) | | 9 (15) | | 1 (50) | |  |
|  | Almost Always | 22 (25) | 22 (27) | | 0 (0) | | 16 (25) | | 16 (26) | | 0 (0) | |  |
| EF5: Worrying | Never | 13 (15) | 9 (11) | | 4 (80) | | 15 (24) | | 14 (23) | | 1 (50) | |  |
|  | Almost Never | 23 (26) | 23 (28) | | 0 (0) | | 9 (14) | | 9 (15) | | 0 (0) | |  |
|  | Sometimes | 45 (52) | 44 (54) | | 1 (20) | | 37 (59) | | 36 (59) | | 1 (50) | |  |
|  | Often | 6 (7) | 6 (7) | | 0 (0) | | 1 (2) | | 1 (2) | | 0 (0) | |  |
|  | Almost Always | 0 (0) | 0 (0) | | 0 (0) | | 1 (2) | | 1 (2) | | 0 (0) | |  |
| SOF1: Getting on with other children/teens | Never | 15 (17) | 14 (17) | | 1 (20) | | 29 (47) | | 28 (47) | | 1 (50) | |  |
|  | Almost Never | 28 (32) | 25 (30) | | 3 (60) | | 12 (19) | | 12 (20) | | 0 (0) | |  |
|  | Sometimes | 23 (26) | 23 (28) | | 0 (0) | | 16 (26) | | 15 (25) | | 1 (50) | |  |
|  | Often | 18 (21) | 17 (21) | | 1 (20) | | 3 (5) | | 3 (5) | | 0 (0) | |  |
|  | Almost Always | 3 (3) | 3 (4) | | 0 (0) | | 2 (3) | | 2 (3) | | 0 (0) | |  |
| SOF2: Other children  /teens not  wanting to be  their friend | Never | 31 (36) | 29 (35) | | 2 (40) | | 33 (54) | | 33 (56) | | 0 (0) | |  |
|  | Almost Never | 24 (28) | 21 (26) | | 3 (60) | | 11 (18) | | 10 (17) | | 1 (50) | |  |
|  | Sometimes | 23 (26) | 23 (28) | | 0 (0) | | 14 (23) | | 13 (22) | | 1 (50) | |  |
|  | Often | 6 (7) | 6 (7) | | 0 (0) | | 1 (2) | | 1 (2) | | 0 (0) | |  |
|  | Almost Always | 3 (3) | 3 (4) | | 0 (0) | | 2 (3) | | 2 (3) | | 0 (0) | |  |
| SOF3: Getting teased  by other  children/teens | Never | 40 (47) | 35 (43) | | 5 (100) | | 40 (65) | | 38 (63) | | 2 (100) | |  |
|  | Almost Never | 21 (24) | 21 (26) | | 0 (0) | | 7 (11) | | 7 (12) | | 0 (0) | |  |
|  | Sometimes | 21 (24) | 21 (26) | | 0 (0) | | 13 (21) | | 13 (22) | | 0 (0) | |  |
|  | Often | 4 (5) | 4 (5) | | 0 (0) | | 1 (2) | | 1 (2) | | 0 (0) | |  |
|  | Almost Always | 0 (0) | 0 (0) | | 0 (0) | | 1 (2) | | 1 (2) | | 0 (0) | |  |
| SOF4: Unable to do things other children/ teens their age can do | Never | 2 (2) | 2 (2) | | 0 (0) | | 0 (0) | | 0 (0) | | 0 (0) | |  |
|  | Almost Never | 4 (5) | 4 (5) | | 0 (0) | | 2 (3) | | 2 (3) | | 0 (0) | |  |
|  | Sometimes | 25 (29) | 22 (27) | | 3 (60) | | 32 (52) | | 31 (53) | | 1 (50) | |  |
|  | Often | 41 (47) | 39 (48) | | 2 (40) | | 12 (20) | | 11 (19) | | 1 (50) | |  |
|  | Almost Always | 15 (17) | 15 (18) | | 0 (0) | | 15 (25) | | 15 (25) | | 0 (0) | |  |
| SOF5: Keeping up  when playing  with other  children/teens | Never | 2 (2) | 2 (2) | | 0 (0) | | 9 (15) | | 8 (14) | | 1 (50) | |  |
|  | Almost Never | 9 (10) | 9 (11) | | 0 (0) | | 5 (8) | | 5 (8) | | 0 (0) | |  |
|  | Sometimes | 23 (26) | 21 (26) | | 2 (40) | | 26 (43) | | 25 (42) | | 1 (50) | |  |
|  | Often | 33 (38) | 30 (37) | | 3 (60) | | 7 (11) | | 7 (12) | | 0 (0) | |  |
|  | Almost Always | 20 (23) | 20 (24) | | 0 (0) | | 14 (23) | | 14 (24) | | 0 (0) | |  |
| SCF1: Paying attention in class | Never | 10 (16) | | 10 (17) | | 0 (0) | | 20 (33) | | 20 (34) | | 0 (0) | |
|  | Almost Never | 14 (23) | | 14 (23) | | 0 (0) | | 8 (13) | | 8 (14) | | 0 (0) | |
|  | Sometimes | 26 (42) | | 24 (40) | | 2 (100) | | 31 (51) | | 29 (49) | | 2 (100) | |
|  | Often | 7 (11) | | 7 (12) | | 0 (0) | | 2 (3) | | 2 (3) | | 0 (0) | |
|  | Almost Always | 5 (8) | | 5 (8) | | 0 (0) | | 0 (0) | | 0 (0) | | 0 (0) | |
| SCF2: Forgetting things | Never | 20 (33) | | 20 (34) | | 0 (0) | | 22 (36) | | 22 (37) | | 0 (0) | |
|  | Almost Never | 27 (44) | | 27 (46) | | 0 (0) | | 14 (23) | | 13 (22) | | 1 (50) | |
|  | Sometimes | 13 (21) | | 12 (20) | | 1 (50) | | 23 (38) | | 22 (37) | | 1 (50) | |
|  | Often | 1 (2) | | 0 (0) | | 1 (50) | | 0 (0) | | 0 (0) | | 0 (0) | |
|  | Almost Always | 0 (0) | | 0 (0) | | 0 (0) | | 2 (3) | | 2 (3) | | 0 (0) | |
| SCF3: Keeping up with school activities | Never | 8 (10) | | 8 (10) | | 0 (0) | | 21 (34) | | 21 (36) | | 0 (0) | |
|  | Almost Never | 13 (16) | | 13 (17) | | 0 (0) | | 7 (11) | | 7 (12) | | 0 (0) | |
|  | Sometimes | 33 (41) | | 31 (40) | | 2 (67) | | 27 (44) | | 26 (44) | | 1 (50) | |
|  | Often | 17 (21) | | 16 (21) | | 1 (33) | | 5 (8) | | 4 (7) | | 1 (50) | |
|  | Almost Always | 9 (11) | | 9 (12) | | 0 (0) | | 1 (2) | | 1 (2) | | 0 (0) | |
| SCF4: Missing school due to not feeling well | Never | 5 (6) | | 5 (6) | | 0 (0) | | 6 (10) | | 6 (10) | | 0 (0) | |
|  | Almost Never | 21 (26) | | 20 (26) | | 1 (33) | | 11 (18) | | 10 (17) | | 1 (50) | |
|  | Sometimes | 38 (48) | | 37 (48) | | 1 (33) | | 37 (61) | | 37 (63) | | 0 (0) | |
|  | Often | 14 (18) | | 13 (17) | | 1 (33) | | 4 (7) | | 3 (5) | | 1 (50) | |
|  | Almost Always | 2 (2) | | 2 (3) | | 0 (0) | | 3 (5) | | 3 (5) | | 0 (0) | |
| SCF5: Missing school to go to the doctor/hospital | Never | 4 (5) | | 3 (4) | | 1 (33) | | 4 (7) | | 4 (7) | | 0 (0) | |
|  | Almost Never | 10 (12) | | 10 (13) | | 0 (0) | | 8 (13) | | 8 (14) | | 0 (0) | |
|  | Sometimes | 56 (70) | | 56 (73) | | 0 (0) | | 45 (74) | | 43 (73) | | 2 (100) | |
|  | Often | 10 (12) | | 8 (10) | | 2 (67) | | 2 (3) | | 2 (3) | | 0 (0) | |
|  | Almost Always | 0 (0) | | 0 (0) | | 0 (0) | | 2 (3) | | 2 (3) | | 0 (0) | |

*Results are presented as number (%)*

*S=RDEB severe (RDEB-S), I=intermediate (RDEB-I),*

*PF=physical functioning; EF=emotional functioning; SF=social functioning; SCF=school functioning*

*^1^ Pediatric Quality of Life Inventory (PedsQL)*
